# Microarray Data Mining and Preliminary Bioinformatics Analysis of Hepatitis D Virus-Associated Hepatocellular Carcinoma

**DOI:** 10.1155/2021/1093702

**Published:** 2021-01-30

**Authors:** Zhe Yu, Xuemei Ma, Wei Zhang, Xiujuan Chang, Linjing An, Ming Niu, Yan Chen, Chao Sun, Yongping Yang

**Affiliations:** ^1^Peking University 302 Clinical Medical School, Beijing 100039, China; ^2^Department of Liver Disease of Chinese PLA General Hospital, The Fifth Medical Center of Chinese PLA General Hospital, Beijing 100039, China; ^3^China Military Institute of Chinese Medicine, The Fifth Medical Centre of Chinese PLA General Hospital, Beijing 100039, China

## Abstract

Several studies have demonstrated that chronic hepatitis delta virus (HDV) infection is associated with a worsening of hepatitis B virus (HBV) infection and increased risk of hepatocellular carcinoma (HCC). However, there is limited data on the role of HDV in the oncogenesis of HCC. This study is aimed at assessing the potential mechanisms of HDV-associated hepatocarcinogenesis, especially to screen and identify key genes and pathways possibly involved in the pathogenesis of HCC. We selected three microarray datasets: GSE55092 contains 39 cancer specimens and 81 paracancer specimens from 11 HBV-associated HCC patients, GSE98383 contains 11 cancer specimens and 24 paracancer specimens from 5 HDV-associated HCC patients, and 371 HCC patients with the RNA-sequencing data combined with their clinical data from the Cancer Genome Atlas (TCGA). Afterwards, 948 differentially expressed genes (DEGs) closely related to HDV-associated HCC were obtained using the R package and filtering with a Venn diagram. We then performed gene ontology (GO) annotation and Kyoto Encyclopedia of Genes and Genomes (KEGG) pathway enrichment analysis to determine the biological processes (BP), cellular component (CC), molecular function (MF), and KEGG signaling pathways most enriched for DEGs. Additionally, we performed Weighted Gene Coexpression Network Analysis (WGCNA) and protein-to-protein interaction (PPI) network construction with 948 DEGs, from which one module was identified by WGCNA and three modules were identified by the PPI network. Subsequently, we validated the expression of 52 hub genes from the PPI network with an independent set of HCC dataset stored in the Gene Expression Profiling Interactive Analysis (GEPIA) database. Finally, seven potential key genes were identified by intersecting with key modules from WGCNA, including 3 reported genes, namely, *CDCA5*, *CENPH*, and *MCM7*, and 4 novel genes, namely, *CDC6*, *CDC45*, *CDCA8*, and *MCM4*, which are associated with nucleoplasm, cell cycle, DNA replication, and mitotic cell cycle. The *CDCA8* and stage of HCC were the independent factors associated with overall survival of HDV-associated HCC. All the related findings of these genes can help gain a better understanding of the role of HDV in the underlying mechanism of HCC carcinogenesis.

## 1. Introduction

Hepatocellular carcinoma (HCC) is the sixth most commonly diagnosed cancer and the fourth leading cause of cancer-related mortality globally [1, 2] and the second in China [3]. More than 80% of all HCC causes are associated with infection with hepatitis B virus (HBV), hepatitis C virus (HCV), and hepatitis delta virus (HDV) [4]. Approximately 292 million people worldwide are chronically infected with HBV, which causes liver injury that can progress to cirrhosis, resulting in HCC, liver failure, and eventually death [5, 6]. The HDV is known as the satellite of HBV and affects 15–20 million people in the world [7]. Concurrent HBV and HDV infections significantly increase both the incidence and mortality of HCC among patients with chronic hepatitis B (CHB) [8]. HDV is a kind of defective RNA virus which uses HBV envelope protein for successful spread in hepatocytes [9]. Although the risk of HCC is thought to be higher when a HBV-infected patient is superinfected with HDV, the molecular mechanisms of carcinogenesis remain unclear [10]. Chronic hepatitis D (CHD) is more severe than any other type of hepatitis, but its carcinogenesis mechanism remains poorly understood. Additionally, it has been found that the intrahepatic HBV DNA levels in patients with HDV-associated HCC and non-HCC cirrhosis are significantly reduced [11]. This phenomenon of HDV-mediated inhibition of HBV replication suggests that the effects of HDV are mediated through a unique molecular mechanism. While HBV and HCV are both included in International Agency for Research on Cancer (IARC) group 1 (high evidence of carcinogenicity to humans), HDV was assigned several years ago to group 3 (not sufficient evidence of carcinogenicity) [12], due to inadequacy to support the contribution of HDV to HBV-induced HCC. Due to the dependency of HDV on HBV, there are still controversies regarding the increased risk of HCC development in chronically HDV-infected patients [4], and the available data on the particular mechanism by which HDV contributes to HCC are sparse. With the development of genomics and other “-omics” disciplines, substantial omics data from HCC specimens have been accumulated [13]. Therefore, researchers have taken advantage of gene ontology (GO) and signal pathway analysis tools to identify and characterize many differentially expressed genes (DEGs).

In the present study, the GSE55092 and GSE98383 mRNA expression profile datasets were retrieved from Gene Expression Omnibus (GEO) online [14, 15]. And the RNA-sequencing data of 371 HCC patients and their clinical data were from the Cancer Genome Atlas (TCGA). We performed DEG analysis between cancerous specimens of HBV-associated HCC and HDV-associated HCC patients and their respective paracancerous specimens using Linear Models for Microarray Data (LIMMA) [16] and other packages implemented in R/Bioconductor [17]. We aimed to investigate potential HDV carcinogenesis mechanisms by PPI network, Gene Expression Profiling Interactive Analysis (GEPIA), and Weighted Gene Coexpression Network Analysis (WGCNA), particularly to screen and identify key genes and pathways to determine their possible role in HCC pathogenesis, and to help determine their mechanism of inhibition of HBV replication and their effects in diagnosis, treatment and prognosis.

## 2. Methods and Materials

### 2.1. Acquisition of Data and Preprocessing

**Figure 1:**
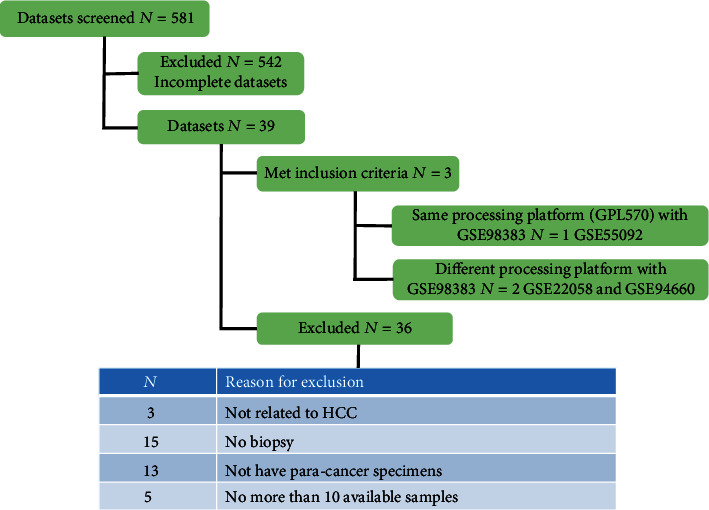
Flow chart of enrolled datasets and availability of datasets.

The RNA-sequencing data and clinical data of 371 HCC patients were downloaded from TCGA (http://cancergenome.nih.gov/. http://cancergenome.nih.gov/). The expression of genes was represented by fragments per kilobase of exon per million fragments mapped (FPKM). Microarray data were available at the National Center for Biotechnology Information (NCBI) Gene Expression Omnibus (GEO, https://www.ncbi.nlm.nih.gov/geo/) database. The inclusion criteria for selection GEO datasets in this study were as follows: (1) hepatocellular carcinoma containing cancer and paracancer tissue; (2) HBsAg positive at least 6 months with serum HBV DNA positive; (3) anti-HDAg positive with serum HDV RNA positive (applies only to filter HDV-associated HCC dataset); and (4) sample size more than 10 with data unbiased. The exclusion criteria were as follows: (1) the second liver cancer, (2) HCV-related HCC, (3) dataset biased, and (4) no paracancer tissue and carcinoma tissue present at the same patients. We searched the GEO database using “Hepatitis D Virus,” “Hepatocellular Carcinoma,” and “Homo sapiens” as keywords, and there were only 2 results, between which only GSE98383 met our criteria for HDV-associated HCC. Similarly, we searched the keywords “Hepatitis B Virus,” “Hepatocellular Carcinoma,” and “Homo sapiens” and obtained 581 search results. The flow chart of screening HBV-associated HCC could be seen in Figure 1, and only GSE55092 was suited for an in-depth study.

Microarray data GSE55092 [18] and GSE98383 [11] were generated using the GPL570 Affymetrix HG-U133 Plus 2.0 Array platform, and we performed the analysis on the data from whole liver tissue. GSE55092 contains 39 cancer specimens and 81 paracancer specimens from 11 HBV-associated HCC patients (average age = 57.7 ± 7.7 years; 10 male patients and 1 female patient). GSE98383 contains 11 cancer specimens and 24 paracancer specimens from 5 HDV-associated HCC patients (average age = 57 ± 3 years, 5 male patients). The comparison of baseline characteristics between patients with HBV-associated HCC and HDV-associated HCC is shown in Table 1.

We downloaded the GSE55092 and GSE98383 datasets, normalized them by the Affy package of the R Bioconductor, and then converted the gene expression profile at the probe level into gene symbol level and removed the duplicated symbols. When numerous probes were mapped to one gene, the average value was defined as the expression level of that gene. According to the description of the uploader, an unsupervised multidimensional scaling (MDS) of all specimens obtained from HBV-associated HCC patients showed a clear separation between two distinct clusters that corresponded to cancer areas and paracancerous areas. A similar separation between two clusters that corresponded to cancerous areas and paracancerous areas was observed for the specimens from HDV-associated HCC patients. Therefore, all the data are available for the identification of DEGs.

### 2.2. Identification of DEGs

DEG analysis refers to the identification of genes with significantly different expression levels between two groups through multiple analysis modes [19]. We performed differential expression analysis using Bayes *t*-statistics from the LIMMA implemented in the R Bioconductor and corrected *p* values for multiple testing using the Benjamini-Hochberg method [20]. We identified the DEGs in primary cancerous specimens of HCC patients by comparing them with paracancerous normal specimens of the same HCC patients. The absolute value of log2-fold change (FC) was set to ≥1.0, and a *p* value of <0.01 was used as the significance criteria, and genes that met these criteria were used for further analysis. The final step is to use Venn diagrams to identify DEGs closely related to HDV-associated HCC [21].

### 2.3. GO Enrichment and Pathway Analysis

DAVID program (https://david.ncifcrf.gov/) [22] is a bioinformatics resource comprising a biological database and a set of annotation and analytical tool that intuitively integrates functional genomic annotations with graphics. In this study, the DEGs were submitted to DAVID for GO [23] and KEGG [24] enrichment analyses, which included biological process (BP), cellular component (CC), molecular function (MF), and related biological metabolic pathways. A *p* value < 0.05 was considered statistically significant.

### 2.4. Weighted Gene Coexpression Network Construction and Module-Clinical Characteristic Associations

We compared DEGs with the genes of 371 HCC patients downloaded from TCGA website. Expression data of the matched genes from TCGA were applied to find gene modules significantly associated with clinical trait (stage of HCC) by WGCNA [25]. In this analysis mode, the soft thresholding acts as the lowest power based on the criterion of approximate scale-free topology [26], and analogous modules would be merged together due to the similarity. The heatmap of module-clinical characteristic relationship could reveal modules significantly associated with clinical characteristics.

### 2.5. Construction of PPI Networks and Module Analysis

The visual protein-to-protein interaction (PPI) networks of DEGs were predicted using the web resource Search Tool for the Retrieval of Interacting Genes (STRING) [27] to search the STRING database (https://string-db.org/), which contains over 5,000 organisms as well as their over 24.6 million proteins and over 2 billion interactions. We correlated the target DEGs with the STRING database and set the significant threshold to the highest confidence level (interaction score ≥ 0.900). Subsequently, we used Cytoscape [28], a software to construct PPI networks and analyze highly interconnected modules using the built-in Molecular Complex Detection (MCODE) clustering algorithm. The parameters were set by default except for the *K*-core value which was equal to 8.

### 2.6. Validation of Module Gene

First, we uploaded the potential genes identified by PPI-network analysis to GEPIA [29] (http://gepia.cancer-pku.cn/, an online server containing TCGA/GTEx datasets) to validate the gene expression consistency between the microarray datasets (GSE55092 and GSE98383) and TCGA/GTEx HCC dataset, setting the threshold parameters as follows: |log2FC| cutoff ≥ 1.0 and *p* value cutoff < 0.01. Afterwards, we performed the overall survival analysis as follows: we divided the patients in TCGA/GTEx dataset into high and low expression groups with the TPM (transcripts per kilobase million) midvalue as a breakpoint; a log-rank test was used to determine significance at *p* < 0.05. Finally, we took the intersection of related genes to the OS of HCC by GEPIA and genes contained in the hub module obtained by WGCNA and got the key genes.

### 2.7. Univariate and Multivariate Cox Proportional Hazards Model Analysis

We performed univariate and multivariate Cox proportional hazards model analysis in patients from TCGA, to find independent factors associated with the overall survival of HCC.

## 3. Result

### 3.1. Comparison of Baseline Characteristics

GSE98383 was the only dataset of HDV-associated HCC that met our criteria, as to the screen of HBV-associated HCC dataset, overall 581 datasets were enrolled and screened for eligibility and 3 datasets met inclusion criteria. Of these 3 datasets, the data processing platform of GSE55092 was GPL570, which was the same with GSE98383, while GSE22058 and GSE94660 were different, so we took GSE55092 to stand for HBV-associated HCC for study. The number of datasets and reasons for exclusion are shown in Figure 1. The HDV-associated HCC patients in GSE98383 had higher levels of alanine aminotransferase (ALT), aspartate aminotransferase (AST), total bilirubin (TB), prothrombin time (PT), platelet counts (PLT), and inflammatory activity grade than the HBV-associated HCC patients in GSE55092, which is consistent with the characteristics of CHD of the most severe hepatitis. On the other hand, there was no significant difference in sex, age, tumor grade, and tumor size between the two groups.

### 3.2. Identification of DEGs

**Figure 2:**
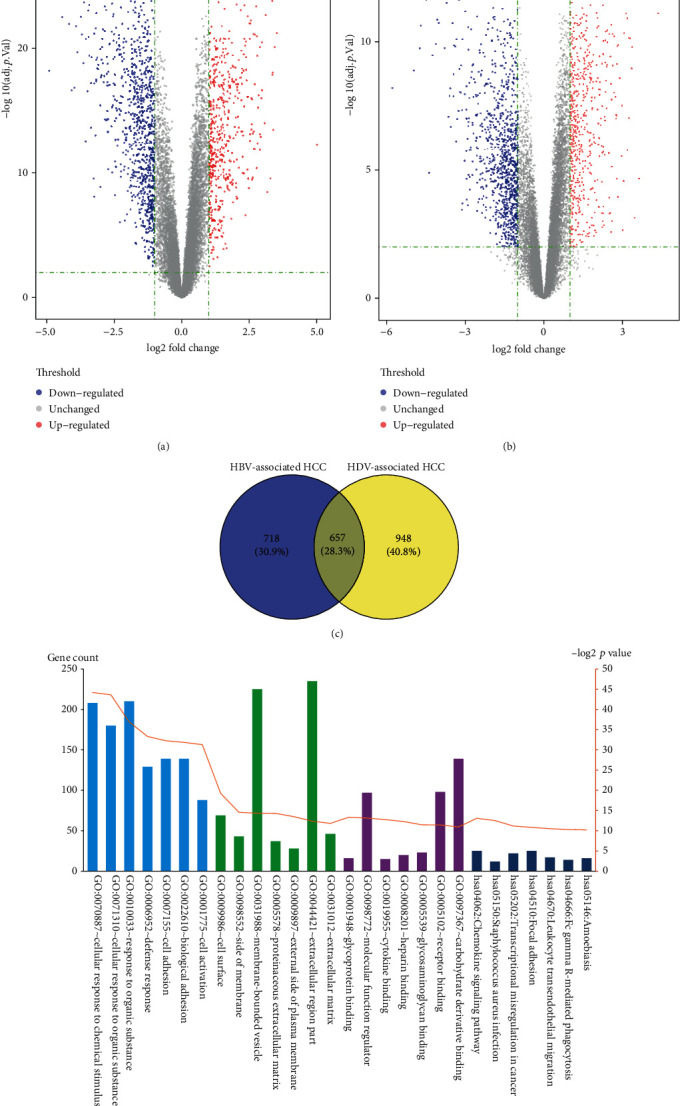
Identification and filtering of DEGs closely related to HDV-associated HCC and their GO and KEGG pathway analysis. (a) Volcano plot of DEGs related to HBV-associated HCC; the DEGs with the top-10 *P* value differences are shown in the plot. (b) Volcano plot of DEGs related to HDV-associated HCC; the DEGs with the top-10 *P* value differences are shown in the plot. (c) The Venn diagram shows the intersection of DEGs between HBV-associated HCC and HDV-associated HCC. The right part (yellow) represents the DEGs closely related to HDV-associated HCC. (d) The GO terms and the KEGG pathways of DEGs significantly enriched in HDV-associated HCC. GO: gene ontology; KEGG: Kyoto Encyclopedia of Genes and Genomes; HCC: hepatocellular carcinoma.

We identified DEGs from the microarray GSE55092 and GSE98383 datasets using the LIMMA package, setting |log2-FC| to ≥1.0 and adjusted *p* value to <0.01 as the criteria. By comparing the cancerous and paracancerous specimens in GSE55092 up to 1,375, DEGs were identified, comprising 518 upregulated and 857 downregulated genes (Table S1). A similar comparison in GSE98383 contains 1,605 DEGs, including 592 upregulated and 1,013 downregulated genes (Table S1). Volcano plots of the GSE55092 and GSE98383 microarrays are shown in Figures 2(a) and 2(b), respectively. We used Venn diagrams to determine the DEGs closely related to HDV-associated HCC (Figure 2(c)), and 948 DEGs (373 upregulated and 582 downregulated) were identified and selected for further analysis (Table S2).

### 3.3. GO Enrichment and Pathway Analysis

In order to further screen HDV-associated HCC potential target genes among these DEGs, GO and pathway analysis were performed on HDV-associated HCC DEGs using *p* value < 0.05 as the threshold (Figure 2(d)). The results are presented in Figure 2(d) and show the TOP-7 GO terms (BP, CC, and MF) and KEGG pathway terms significantly enriched in the DEGs. Additionally, the TOP-5 annotations of the DEGs are shown in Table 2. In the BP series, the DEGs were mostly enriched for genes related to the cellular response to chemical stimulus and organic substance, defense response, and cell adhesion. In the CC series, the DEGs were primarily enriched for genes involved in cell surface, side of membrane, membrane-bounded vesicle, external side of plasma membrane, and proteinaceous extracellular matrix. In the MF series, the DEGs were predominantly enriched for genes associated with glycoprotein binding, molecular function regulator, cytokine binding, heparin binding, and glycosaminoglycan binding. In the KEGG pathway series, the enrichment for DEGs was mainly in the chemokine signaling pathway, Staphylococcus aureus infection, transcriptional misregulation in cancer, focal adhesion, and leukocyte transendothelial migration.

### 3.4. Weighted Gene Coexpression Network Construction and Module-Clinical Characteristics

**Figure 3:**
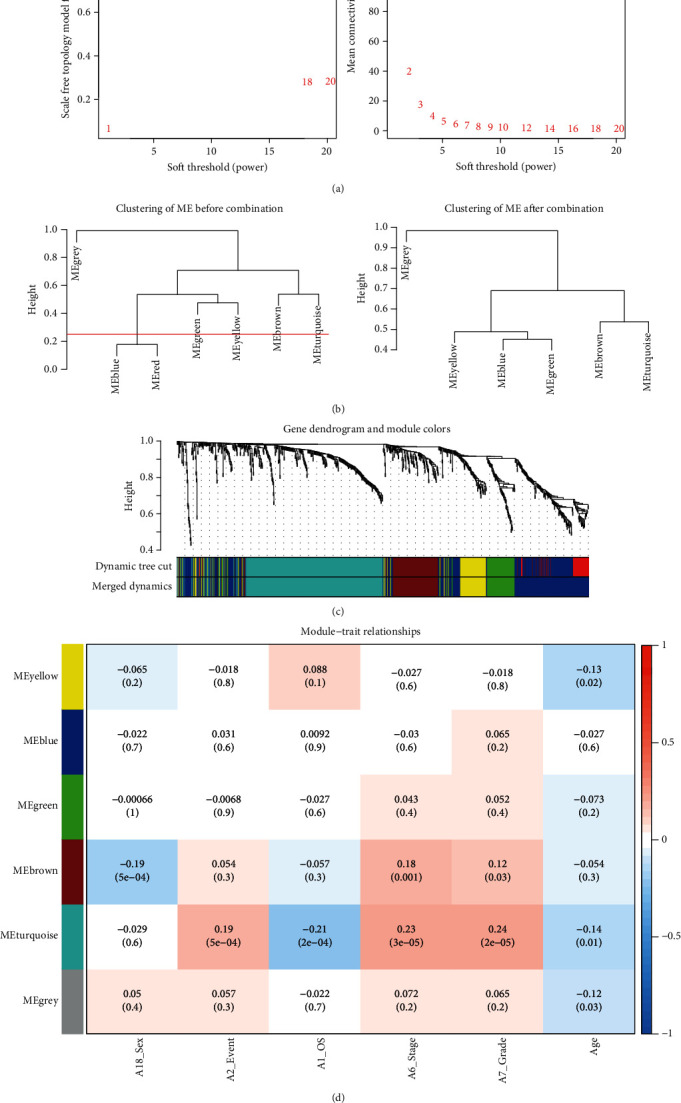
The processing steps of WGCNA. (a) Analysis of the soft thresholding power (*β* = 3). (b) MEblue and MEred merged together due to the similarity (the height = 0.25). (c) Gene dendrogram and module colors; the MEturquoise contained the most DEGs (*n* = 320). (d) Heatmap of module-trait relationships. The MEturquoise was the most significantly associated with event, OS, stage, grade, and age. WGCNA: Weighted Gene Coexpression Network Analysis.

We compared 948 DEGs with the genes of 371 samples downloaded from TCGA datasets, matched a total of 883 genes, and performed WGCNA. As shown in Figures 3(a), 3(b), and 3(c), the soft thresholding power *β* was set to 3, and MEblue and MEred were merged together due to the similarity (the height = 0.25). Then, we found five coexpressed gene modules. The MEturquoise contained the most DEGs with the number of 320. The five modules and contents are stored in supplementary material Table S3. Next, we further analyzed these modules with clinical characteristics (sex, event, OS, stage, grade, and age). Obviously, the MEturquoise module was significantly associated with event (correlation coefficients (*r*) = 0.19, *p* < 0.001), OS (*r* = −0.21, *p* < 0.001), stage (*r* = 0.23, *p* < 0.001), grade (*r* = 0.24, *p* < 0.001), and age (*r* = −0.14, *p* = 0.01) (Figure 3(d)).

### 3.5. Construction of PPI Networks and Gene Module Analysis

**Figure 4:**
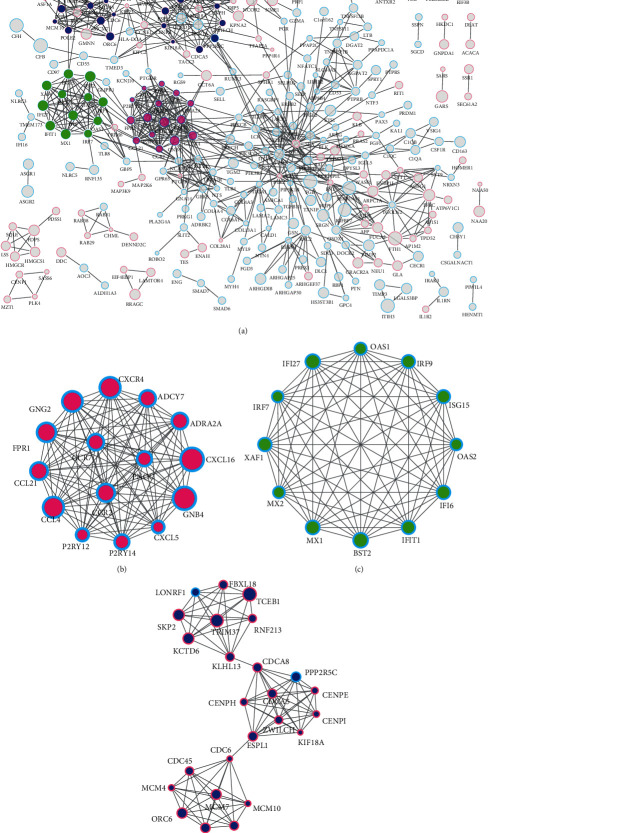
PPI networks and the top-3 significant modules (module 1-3). (a) PPI networks constructed with the DEGs closely related to HDV-associated HCC. The red border indicates upregulation, the blue border indicates downregulation, the pink core represents module 1, the green core represents module 2, the dark blue core represents module 3, and the size of the circle represents the relative expression level of the genes; (b) module 1, the DEGs in module 1 are all downregulated; (c) module 2, the DEGs in module 2 are all downregulated; (d) module 3, the DEGs in module 3 are upregulated except for *PPP2R5C* and *LONRF1*. PPI: protein-to-protein interaction; DEGs: differentially expressed genes.

We uploaded the DEGs onto the STRING online tool and analyzed them with the Cytoscape software. We then selected 353 nodes and 939 edges with the highest confidence (scores > 0.900) to construct the PPI networks (Figure 4(a)). Then, the MCODE plugin filtered out three important gene modules. Genes within module 1 and module 2 are comprised of downregulated genes, while module 3 is comprised of upregulated genes, except for *PPP2R5C* and *LONRF1*. Module 1 contains 15 nodes and 105 edges (Figure 4(b)), which are mainly related to G protein-coupled receptor signaling pathway (BP), plasma membrane (CC), G protein-coupled receptor binding (MF), and chemokine signaling pathway (KEGG) (Table 3). Module 2 contains 12 nodes and 66 edges (Figure 4(c)), which are primarily related to type I interferon signaling pathway (BP), cytosol (CC), 2′-5′-oligoadenylate synthetase activity (MF), and hepatitis C (KEGG) (Table 4). Module 3 contains 25 nodes and 94 edges (Figure 4(d)), which are predominantly related to the mitotic cell cycle process (BP), chromosomal part (CC), DNA replication origin binding (MF), and cell cycle (KEGG) (Table 5).

### 3.6. Validation of Module Genes

**Figure 5:**
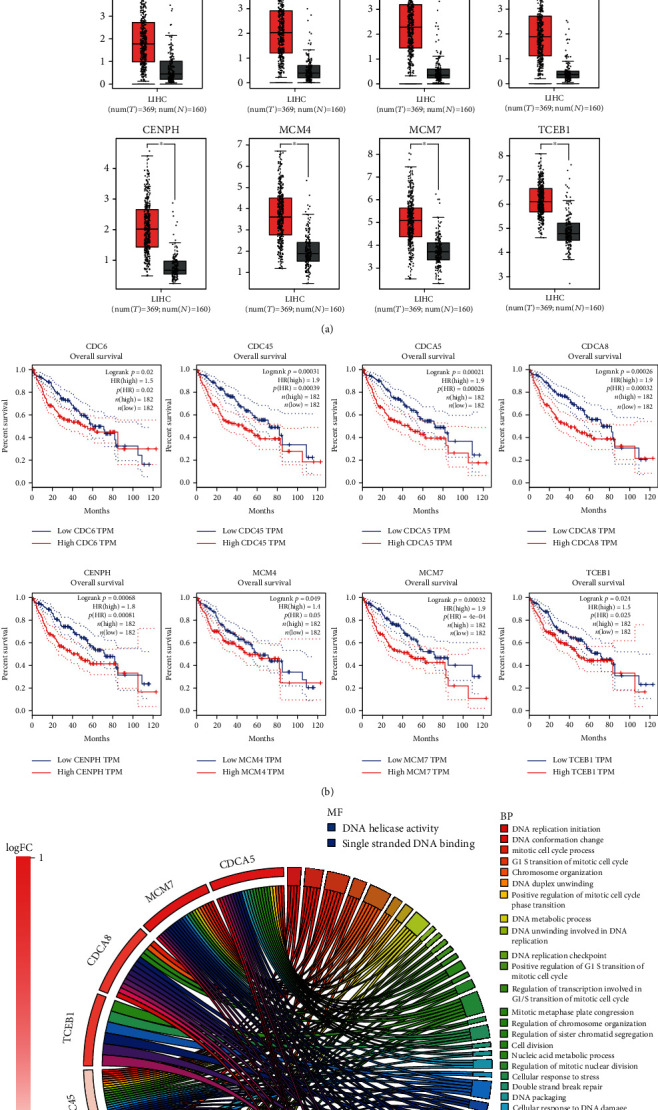
Validation of the expression data and survival curve of hub genes from the 3 modules using the GEPIA database and functional and pathway enrichment analysis. (a) The box plots that verify whether the expression of these DEGs is consistent with that in the LIHC datasets. Among the downregulated genes, *CCL21* and *FPR1* (module 1) as well as *XAF1* (module 2) are consistent with the HCC datasets, while *IFI6*, *IFI27*, and *ISG15* (module 2) are not. All 8 upregulated genes (module 3) are consistent with the LIHC datasets. (b) The genes are associated with overall survival whose expression is consistent with that in the LIHC datasets. All 8 upregulated genes are from module 3. (c) The chord diagram showing GO terms and KEGG pathway enrichment with the 8 hub genes involved. (d) The intersection of these 8 genes from PPI network analysis and 320 genes contained in module MEturquoise obtained by WGCNA, 7 potential key genes in the middle part. HCC: hepatocellular carcinoma; GO: gene ontology; KEGG: Kyoto Encyclopedia of Genes and Genomes; BP: biological process; CC: cellular component; MF: molecular function; PPI: protein-to-protein interaction; WGCNA: Weighted Gene Coexpression Network Analysis.

We compared the gene expression changes between the three module genes of HDV-associated HCC (a total of 52 genes) and the validation HCC (TCGA/GTEx) datasets in the GEPIA website to verify whether their expression in both datasets is consistent. We noticed that *CCL21* and *FPR1* in module 1 as well as *XAF1* in module 2 were downregulated in tumors compared to normal specimens in the HCC datasets, which is in accordance with the HDV-associated HCC specimens. However, *IFI6*, *IFI27*, and *ISG15* in module 2, which were downregulated in cancerous specimens compared to paracancerous normal specimens, were conversely expressed in HCC datasets. The genes in module 3, including *CDC6*, *CDC45*, *CDCA5*, *CDCA8*, *CENPH*, *MCM4*, *MCM7*, and *TCEB1*, were upregulated in tumor compared to normal specimens in the HCC datasets, which is consistent with the HDV-associated HCC patients. All the box plots comparing gene expression are shown in Figure 5(a). For further verification, 11 genes whose gene expression trends are consistent with HCC datasets were selected and used to conduct overall survival analysis. In Figure 5(b), there were the upregulated genes (*CDC6*, *CDC45*, *CDCA5*, *CDCA8*, *CENPH*, *MCM4*, *MCM7*, and *TCEB1*) which are associated with a lower survival rate in the high expression group than in the low expression group.

The reason for excluding *CCL21*, *FPR1*, *IFI6*, *IFI27*, *ISG15*, and *XAF1* is that their *p* value did not comply with the standards or the opposite gene expression. All the 8 retained potential genes are related to the nucleoplasm, and most of them are related to the mitotic cell cycle process, cell cycle, and DNA replication (Figure 5(c)). Taken together, the intersection of the above validated 8 genes and 320 genes in MEturquoise module by WGCNA, the potential 7 key genes (*CDC6*, *CDC45*, *CDCA5*, *CDCA8*, *CENPH*, *MCM4*, and *MCM7*) were found (Figure 5(d)).

### 3.7. Identification of Independent Factors of Overall Survival of HCC

We performed the univariate analysis in 371 patients from TCGA and found that *CDCA8*, stage, *CDC45*, *CDC6*, *CDCA5*, *MCM4*, *CENPH*, *MCM7*, sex, and age were significantly associated with OS of HCC. The multivariate Cox proportional hazards model showed that *CDCA8* and stage of HCC were independent factors of OS of HCC (Table 6).

## 4. Discussion

As the virus causing the most severe type of hepatitis, HDV affects 15-20 million people worldwide, but its specific pathogenic mechanism remains unclear. Accordingly, we undertook to find potential genes and pathways involved in the pathogenesis of this disease through text mining to help explain the underlying carcinogenic mechanism of HDV as well as the HBV inhibitory mechanism.

In this study, we compared cancerous and paracancerous specimens of patients suffering from HBV or HDV-associated HCC with the aim of identifying potential genes closely related to HDV-associated HCC. The study identified 373 upregulated DEGs and 582 downregulated DEGs. These DEGs were subjected to GO and KEGG annotation and enrichment analyses. In addition, we constructed PPI networks and sorted out 353 nodes with 939 edges, from which the three most significant modules were selected and 52 central nodes/genes were selected for validation using the GEPIA database. In the module confirmed by the WGCNA that was significantly associated with clinical features including event, OS, stage, grade, and age, only *CDC6*, *CDC45*, *CDCA5*, *CDCA8*, *CENPH*, *MCM4*, and *MCM7* were consistent with genes identified by the PPI network, which were found to be significantly correlated with nucleoplasm, cell cycle, DNA replication, and mitotic cell cycle. Univariate and multivariate Cox proportional hazards model analysis showed the stage of HCC and CDCA8 are the independent factors associated with the OS of HCC.

Cell division cycle 6 (CDC6) is thought to be significantly associated with pancreatic cancer and colorectal cancer (CRC) [30]. It has a pivotal role in regulating the process of DNA replication as well as tumorigenesis; its overexpression could interfere with the expression of tumor suppressor genes (*INK4/ARF*) through the mechanism of epigenetic modification [31]. During the S phase of DNA replication in eukaryotic cells, cell division cycle 45 (CDC45) is an essential component of CMG (CDC45–MCM–GINS) helicase. CDC45 acts as a hubprotein, significantly upregulated in cancerous tissues from CRC and non-small-cell lung cancer (NSCLC) patients, and promotes tumor progression [32]. It is established that the MCM4/6/7 (minichromosome maintenance complex component 4/6/7) hexamer complex acts as a DNA helicase. Additionally, in endometrial cancer and skin cancer studies, it was found that MCM4 mutations may affect the interaction with MCM7, thereby disrupting the stability of the MCM4/6/7 complex [33]. In addition, MCM4 is also a member of significant predictors of poor prognosis in CRC patients [34]. Moreover, MCM7 is also a promising biomarker for early diagnosis of gastric cancer and even a predictor of meningioma recurrence after surgery [35, 36]. Another study found that high expression of MCM7 may be involved in the progression of HCC through the MCM7-cyclin D1 pathway, and MCM7 may serve as a prognostic marker for patients with HCC [37]. The cell division cycle-associated protein 5 (CDCA5) is a member of the CDCA family that comprises CDCA1-8. It plays a crucial role as a regulator of sister-chromatid cohesion and separation during cell division, and its upregulation has been shown to be associated with various cancers, including breast cancer, esophageal squamous cell carcinoma, CRC, and HCC [38, 39]. Also, a study found that the activation of the ERK and AKT pathways may be involved in the regulation of HCC cell proliferation by CDCA45 [39]. CDCA8 is an essential regulator of mitosis, and its overexpression is significantly associated with bladder cancer, cutaneous melanoma, and the progression and prognosis of breast cancer [40, 41]. Centromere protein H (CENPH) is considered to be an essential part of the active centromere complex, and its overexpression is highly related to poor prognosis in renal cell carcinoma, nasopharyngeal carcinoma, CRC, and HCC [42, 43]. Another study found that CENPH may promote the proliferation of HCC through the mitochondrial apoptosis pathway [43].

Most of the abovementioned genes are significantly associated with the cell cycle and DNA replication, and their overexpression may affect the replication of HBV DNA, thereby promoting the unique phenomenon of HDV inhibits HBV replication. All the findings related to these genes may also help us understand the mechanisms of HDV-induced liver injury and HCC. In the future, we will further verify those genes' function by performing animals, cells, and clinical trials.

## 5. Conclusions

In summary, 7 potential candidate genes closely related to HDV-associated HCC were identified in this study. Through comparative analysis with previous studies, these genes were found to be involved in many pathways related to tumorigenesis which provided clues to elucidate the mechanism of hepatitis D virus-induced HCC or its unique molecular mechanism in the inhibition of HBV replication. However, additional in-depth molecular biological research on these candidate genes closely related to HDV-associated HCC is necessary to confirm their functions.

## Figures and Tables

**Table 1:** Comparison of baseline characteristics between patients with HBV-associated HCC and HDV-associated HCC.

	HBV-associated HCC	HDV-associated HCC	*p*
Patients	11	5	—
Age (years old)	57.7 ± 7.7	57 ± 3	0.849
Male (%)	10 [90.9]	5 [100]	1.000
ALT (U/L)	36.18 ± 17.8	87 ± 25	0.000
AST (U/L)	39.09 ± 17.0	82 ± 21	0.001
GGT (U/L)	93.9 ± 83.56	98 ± 22	0.917
PT (INR)	1.13 ± 0.14	1.4 ± 0	0.001
TB (mg/dL)	0.88 ± 0.47	2.3 ± 1.3	0.005
PLT (10^3^/mL)	15.381 ± 9.373	101.4 ± 16.1	0.000
*Liver pathology*			
Activity grade	5.75 ± 3.06	9.1 ± 1.1	0.034
Fibrosis stage	5.1 ± 1.56	6.0 ± 0.0	0.226
F5/F6	9	5	
*Tumor grade*			0.107
G2	7	1	
G3	3	4	
G4	1	0	
*Tumor size*			0.407
<2 cm	4	0	
≥2 and ≤3 cm	4	3	
>3 cm	3	2	
Serum HDV RNA positive, no.	0	5	—
Serum HBV DNA positive, no.	11	5	—

ALT: alanine aminotransferase; AST: aspartate aminotransferase; GGT: *γ*-glutamyl transferase; TB: total bilirubin; PT: prothrombin time; PLT: Platelets.

**Table 2:** The top five annotations in GO and KEGG enrichment analysis of the DEGs.

Category	Term	Count	*p* value
GOTERM_BP_FAT	GO:0070887~cellular response to chemical stimulus	208	4.96*E* − 14
GOTERM_BP_FAT	GO:0071310~cellular response to organic substance	180	7.46*E* − 14
GOTERM_BP_FAT	GO:0010033~response to organic substance	210	7.89*E* − 12
GOTERM_BP_FAT	GO:0006952~defense response	129	9.24*E* − 11
GOTERM_BP_FAT	GO:0007155~cell adhesion	139	1.95*E* − 10
GOTERM_CC_FAT	GO:0009986~cell surface	69	1.59*E* − 06
GOTERM_CC_FAT	GO:0098552~side of membrane	43	4.16*E* − 05
GOTERM_CC_FAT	GO:0031988~membrane-bounded vesicle	225	4.83*E* − 05
GOTERM_CC_FAT	GO:0005578~proteinaceous extracellular matrix	37	4.95*E* − 05
GOTERM_CC_FAT	GO:0009897~external side of plasma membrane	28	8.75*E* − 05
GOTERM_MF_FAT	GO:0001948~glycoprotein binding	16	9.68*E* − 05
GOTERM_MF_FAT	GO:0098772~molecular function regulator	97	1.11*E* − 04
GOTERM_MF_FAT	GO:0019955~cytokine binding	15	1.44*E* − 04
GOTERM_MF_FAT	GO:0008201~heparin binding	20	2.02*E* − 04
GOTERM_MF_FAT	GO:0005539~glycosaminoglycan binding	23	3.53*E* − 04
KEGG_PATHWAY	hsa04062: Chemokine signaling pathway	25	1.16*E* − 04
KEGG_PATHWAY	hsa05150: Staphylococcus aureus infection	12	1.73*E* − 04
KEGG_PATHWAY	hsa05202: Transcriptional misregulation in cancer	22	4.38*E* − 04
KEGG_PATHWAY	hsa04510: Focal adhesion	25	5.49*E* − 04
KEGG_PATHWAY	hsa04670: Leukocyte transendothelial migration	17	6.91*E* − 04

**Table 3:** Functional and pathway enrichment of module 1 genes.

Category	Term	Count	*p* value	Genes
GOTERM_BP_FAT	G protein-coupled receptor signaling pathway	14	2.79*E* − 13	ADCY7, ADRA2A, CCL21, CCL4, CCR2, CCR7, CXCL16, CXCL5, CXCR4, FPR1, GNG2, P2RY12, P2RY14, PNOC
GOTERM_BP_FAT	Cell chemotaxis	8	2.16*E* − 10	CCL21, CCL4, CCR2, CCR7, CXCL16, CXCL5, CXCR4, FPR1
GOTERM_BP_FAT	Chemokine-mediated signaling pathway	6	6.75*E* − 09	CCL21, CCL4, CCR2, CCR7, CXCL5, CXCR4
GOTERM_CC_FAT	Plasma membrane	11	0.0205	ADCY7, ADRA2A, CCR2, CCR7, CXCL16, CXCR4, FPR1, GNG2, P2RY12, P2RY14, PNOC
GOTERM_CC_FAT	External side of plasma membrane	3	0.0205	CCR2, CCR7, P2RY12
GOTERM_CC_FAT	Side of membrane	4	0.0205	CCR2, CCR7, GNG2, P2RY12
GOTERM_MF_FAT	G protein-coupled receptor binding	7	7.55*E* − 08	ADRA2A, CCL21, CCL4, CCR2, CXCL16, CXCL5, PNOC
GOTERM_MF_FAT	Chemokine receptor binding	5	7.55*E* − 08	CCL21, CCL4, CCR2, CXCL16, CXCL5
GOTERM_MF_FAT	Chemokine activity	4	2.30*E* − 06	CCL21, CCL4, CXCL16, CXCL5
KEGG_PATHWAY	Chemokine signaling pathway	10	1.15*E* − 15	ADCY7, CCL21, CCL4, CCR2, CCR7, CXCL16, CXCL5, CXCR4, GNB4, GNG2
KEGG_PATHWAY	Cytokine-cytokine receptor interaction	7	1.67*E* − 08	CCL21, CCL4, CCR2, CCR7, CXCL16, CXCL5, CXCR4
KEGG_PATHWAY	Circadian entrainment	3	0.00092	ADCY7, GNB4, GNG2

**Table 4:** Functional and pathway enrichment of module 2 genes.

Category	Term	Count	*p* value	Genes
GOTERM_BP_FAT	Type I interferon signaling pathway	12	2.85*E* − 27	BST2, IFI27, IFI6, IFIT1, IRF7, IRF9, ISG15, MX1, MX2, OAS1, OAS2, XAF1
GOTERM_BP_FAT	Defense response to virus	9	1.15*E* − 14	BST2, IFIT1, IRF7, IRF9, ISG15, MX1, MX2, OAS1, OAS2
GOTERM_BP_FAT	Negative regulation of viral genome replication	5	3.48*E* − 09	BST2, IFIT1, ISG15, MX1, OAS1
GOTERM_CC_FAT	Cytosol	10	0.004	BST2, IFIT1, IRF7, IRF9, ISG15, MX1, MX2, OAS1, OAS2, XAF1
GOTERM_CC_FAT	Mitochondrion	6	0.0067	IFI27, IFI6, MX1, MX2, OAS1, XAF1
GOTERM_CC_FAT	Cytoplasmic part	12	0.0067	BST2, IFI27, IFI6, IFIT1, IRF7, IRF9, ISG15, MX1, MX2, OAS1, OAS2, XAF1
GOTERM_MF_FAT	2′-5′-Oligoadenylate synthetase activity	2	0.00028	OAS1, OAS2
GOTERM_MF_FAT	Double-stranded RNA binding	2	0.0229	OAS1, OAS2
KEGG_PATHWAY	Hepatitis C	5	1.72*E* − 07	IFIT1, IRF7, IRF9, OAS1, OAS2
KEGG_PATHWAY	Measles	5	1.72*E* − 07	IRF7, IRF9, MX1, OAS1, OAS2
KEGG_PATHWAY	Influenza A	5	1.92*E* − 07	IRF7, IRF9, MX1, OAS1, OAS2

**Table 5:** Functional and pathway enrichment of module 3 genes.

Category	Term	Count	*p* value	Genes
GOTERM_BP_FAT	Mitotic cell cycle process	16	2.69*E* − 16	CDC45, CDC6, CDCA5, CDCA8, CENPE, DBF4, ESPL1, KIF18A, MCM10, MCM4, MCM7, ORC6, POLE2, PPP2R5C, SKP2, ZWILCH
GOTERM_BP_FAT	Cell cycle	17	6.32*E* − 13	CDC45, CDC6, CDCA5, CDCA8, CENPE, DBF4, ESPL1, KIF18A, KLHL13, MCM10, MCM4, MCM7, ORC6, POLE2, PPP2R5C, SKP2, ZWILCH
GOTERM_BP_FAT	G1/S transition of mitotic cell cycle	9	4.80*E* − 12	CDC45, CDC6, DBF4, MCM10, MCM4, MCM7, ORC6, POLE2, SKP2
GOTERM_CC_FAT	Chromosomal part	13	5.18*E* − 10	CDC45, CDCA5, CDCA8, CENPE, CENPH, CENPI, KIF18A, MCM10, MCM7, ORC6, POLE2, PPP2R5C, ZWILCH
GOTERM_CC_FAT	Chromosome, centromeric region	8	3.39*E* − 09	CDCA5, CDCA8, CENPE, CENPH, CENPI, KIF18A, PPP2R5C, ZWILCH
GOTERM_CC_FAT	Intracellular nonmembrane-bounded organelle	18	1.05*E* − 06	CDC45, CDC6, CDCA5, CDCA8, CENPE, CENPH, CENPI, ESPL1, KCTD6, KIF18A, MCM10, MCM7, ORC6, POLE2, PPP2R5C, RNF213, SKP2, ZWILCH
GOTERM_MF_FAT	DNA replication origin binding	3	0.00011	CDC45, MCM10, ORC6
GOTERM_MF_FAT	Single-stranded DNA binding	4	0.00037	CDC45, MCM10, MCM4, MCM7
GOTERM_MF_FAT	DNA helicase activity	3	0.00069	CDC45, MCM4, MCM7
KEGG_PATHWAY	Cell cycle	8	8.55*E* − 11	CDC45, CDC6, DBF4, ESPL1, MCM4, MCM7, ORC6, SKP2
KEGG_PATHWAY	DNA replication	3	0.00022	MCM4, MCM7, POLE2
KEGG_PATHWAY	Ubiquitin-mediated proteolysis	4	0.00024	KLHL13, SKP2, TCEB1, TRIM37

**Table 6:** Univariate and Multivariate Cox proportional hazards model analysis of overall survival of HCC.

Variables	Univariate analysis			Multivariate analysis		
	HR	95% CI	*p* value	HR	95% CI	*p* value
CDCA8	1.11	1.08-1.15	<0.001	1.10	1.06-1.14	<0.001
Stage I			<0.001			0.019
Stage II	1.43	0.88-2.34	0.151	1.23	0.75-2.02	0.416
Stage IIIA	2.68	1.70-4.21	<0.001	2.11	1.31-3.39	0.002
Stage IIIB	2.87	1.02-8.04	0.045	1.84	0.62-5.44	0.273
CDC45	1.13	1.07-1.19	<0.001			
CDC6	1.10	1.05-1.15	<0.001			
CDCA5	1.09	1.05-1.13	<0.001			
MCM4	1.06	1.04-1.09	<0.001			
CENPH	1.18	1.08-1.28	<0.001			
MCM7	1.01	1.00-1.01	0.029			
Gender (F vs. M)	0.82	0.57-1.16	0.257			
Age (years)	1.01	0.99-1.02	0.403			

F: female; M: male.

## Data Availability

The datasets used to support the findings of this study are available from GEO (https://www.ncbi.nlm.nih.gov/geo/) and TCGA (https://portal.gdc.cancer.gov/).

## Supplementary Materials

Supplementary Materials Table S1: DEGs from microarray datasets GSE55092 and GSE98383. Table S2: 948 DEGs related to HDV-associated HCC including 373 upregulated and 582 downregulated genes. Table S3: the five modules and contents obtained by WGCNA.
