# Supplementary material for: Microarray Data Mining and Preliminary Bioinformatics Analysis of Hepatitis D Virus-Associated Hepatocellular Carcinoma
Source: Biomed Res Int. 2021 Jan 30;2021:1093702. doi: 10.1155/2021/1093702 (PMC7867452; doi:10.1155/2021/1093702)
Supplement: Supplementary Materials — Table S1: DEGs from microarray datasets GSE55092 and GSE98383. Table S2: 948 DEGs related to HDV-associated HCC including 373 upregulated and 582 downregulated genes. Table S3: the five modules and contents obtained by WGCNA. [file 1093702.f1.zip › Table S1.docx]

| Table S1: DEGs from Microarray GSE55092 and GSE98383 | | |
| --- | --- | --- |
| Microarray | DEGs | Gene name |
| GSE55092 | Upregulated  genes | SLC7A11, HMMR, CCDC34, FAM189B, STEAP2, CAP2, TRIM45, TOP2A, SPC24, AP000525.9, FAM83D, ASPM, C1orf43, NCAPG, SULT1C2, CENPF, NDC80, CDK1, MRAP2, SPATS2, TK1, LPL, IGF2BP3, COG2, SMPX, RBM24, PI15, UGGT1, CDCA3, DUT, SPDL1, RACGAP1, MELK, SLC16A1-AS1, MCM8, PSMD4, PBK, HDAC11, ANLN, HIST1H3E, ITGA6, CCNB1, TMEM106C, ROBO1, LOC344887, FOXRED2, COA6, PTTG1, TARBP1, CDKN3, MAP2, CD109, KIF20A, LOC401068, AKR1C3, LL22NC03-N14H11.1, RRM2, DLG5, CCNB2, CENPW, CR936796, UBE2C, CPPED1, BUB1B, LOC389834, S100A10, CEP41, THY1, EPHB2, PRR11, STIL, STXBP6, RP11-295G20.2, GNAL, LOC284926, CKAP4, CENPU, SAC3D1, AURKA, DNAJC6, NUSAP1, PRC1, ZKSCAN3, FAM133A, ZFP41, EML6, NOX4, CCAR2, FANCD2, KIF18B, NRCAM, GPR158, TBC1D16, NKAIN2, PEG10, SH3RF2, KIF2C, PACSIN2, AGPAT4, FLYWCH1, SERPINI1, SLC7A6, DTL, ZNF174, PTGFRN, NEB, FLVCR1, GINS1, RNF43, LRRC45, IRX3, CDKN2C, PIEZO2, VASH2, SPSB2, E2F8, CTC-338M12.4, STMN1, KIF11, ZSWIM5, TCF19, KIF14, TTK, LOC81691, TRIM16, E2F7, DLGAP5, ASAP2, ZNF703, LOC284513, LOC101927699, KIF4A, PSPH, ST8SIA6-AS1, C9orf152, FANCI, SMC2, SP5, MTFR2, BIRC5, ITGA2, DUXAP10, CCDC88A, COLCA2, DNAH12, ZNF595, CCNA2, RP11-196G18.24, NUF2, TP53I3, MKI67, GNAI1, ZIC2, CDC25C, PRKAA2, SFN, COX7B2, GBAP1, DEPDC1, LINC00348, CDC20, COL24A1, RAD51AP1, FERMT1, CPD, DYNC1I1, CDKN2A, TOMM40L, SLC22A4, APOBEC3B, IQGAP3, FOXM1, PCDH17, TBCE, H2AFX, CENPK, ASXL2, FIGNL1, LINC00328, ARHGEF28, SLC6A13, GGPS1, PTPRG, C1orf112, FKBP11, CEP152, TALDO1, ISX, LOC101930114, LCN2, STAU2, TAPT1-AS1, UBAP2L, RP11-1109F11.5, HJURP, TKT, CNKSR2, BUB1, KIAA0101, EHMT2, RGS5, ZBED8, GJC1, EZH2, CDK5RAP2, HTATIP2, COL7A1, BRCA1, CNTNAP4, DTNA, USH1C, TMEM74B, STEAP1, ESM1, SGOL2, UNKL, HEXA, GPSM2, LOC100134822, RP11-180N14.1, PCAT6, EFCAB2, MLEC, SLC44A5, TRIP13, HOXA13, TRAM1L1, RRAGD, LYPD8, ICK, CCDC170, LOC100507053, TCFL5, TMEM64, LOC100506844, ARHGAP18, PIR, FGF13, DKK1, CDC7, C6orf62, HOXA3, LEF1, FSTL5, CTSC, MCM6, ECT2, MCM3AP-AS1, CHRM3, SLC6A2, TIGD1, STRA13, SLCO6A1, ACTN2, LOC101926913, CTNNA2, LINC01419, KITLG, LOC728392, TSPAN5, CSMD1, PRR5L, TXNRD1, GPC3, LOC100505851, NEK2, PHYHIPL, ABCB6, SPX, COL15A1, KNTC1, LOC101928000, LOC100505498, TUBG1, SLC1A4, DKK3, GLUL, SPINK5, SSX3, RP5-1074L1.4, BC041025, DNM3, EME1, SSX1, ZNF385D, HRCT1, MAD2L1, REG3A, CENPM, C1QTNF3, NCAPD2, C1orf85, DSCC1, EID3, ARHGAP44, KIAA1462, TTC30B, SRXN1, CRNDE, LINC01004, ANKFN1, LINC00094, RASSF4, DQ592442, ILDR2, PFKFB2, MCM3, TRIM71, ZWINT, ZNF280C, SHCBP1, ACSL6, RP11-394I13.2, FEN1, TRIB2, LOC100287497, THBS4, CKAP2, PRIM1, AMACR, POLN, LOX, PRKD1, CDKN2B, RMST, TPX2, LOC101928728, RP11-196G18.23, CRIP3, RAD54L, KCNE3, PTGR1, LAMA3, SNTG1, IMPAD1, GAD1, CEMIP, GCNT3, TAB3, BRIP1, LAMC1, RBP7, DCAF4L2, GSTA4, ODAM, KIF15, SPP1, LINC01139, STRIP2, TMEM44-AS1, PODXL, CDK5, C5orf66, RALGPS1, CTB-92J24.2, CCNE2, SGK223, CHEK1, SLC38A6, OSR2, LOC102723493, CTD-2035E11.5, INTU, MUC15, KCNU1, IQCH, SLC22A11, RP11-401P9.4, HS2ST1, RFC4, UHRF1, CA12, EPS8L3, SPC25, NKD1, MECOM, EYA4, MAGEA1, TPTE, KCNJ5, MTR, ATP6V0E2, SMYD3, TTC39A, TERT, ASPSCR1, KIAA1244, TPR, CDK6, LOC286382, FRMD3, BAMBI, MCM2, EGF, LPPR1, NLRP11, TNFRSF19, TRPM3, FGF14, LOC646736, C16orf95, HOXA10, HHIPL2, COL5A2, ALDH3A1, SPINK1, PGC, TBX3, SLC45A2, MAGEA6, HS3ST4, ACSL4, GULP1, RNF157, TRIM59, AXIN2, RHBG, TOX3, GREB1, SLC2A5, PRLR, LYZ, LMOD1, LOC285556, SULT4A1, MAGEC1, SPARCL1, CACNA2D3, GAGE3, GGH, RIMS2, CELF6, MAGEC2, ZIC1, LINC00607, LOC727916, FAM169A, AKR1B10, SMEK3P, ERV3-2, NQO1, MAGEA12, PAGE4, LYPD1, B3GNT5, LOC101928622, PLCB1, ANKRD29, SH3GL3, SGCE, G6PD, AGBL3, MYEF2, FCGBP, C21orf37, KCNH8, PLAG1, CABYR, RHOBTB1, CEP55, FKBP1B, ASB4, LINC01021, ZNRF3, MSH2, ERVMER34-1, TMED6, DHRS2, COL4A1, SCPEP1, CD36, COL4A2, DEPDC1B, TRIM55, AVIL, HSPB1, LOC102723847, EDIL3, PKD1L2, FGF2, DOCK3, WNT5A, GOLM1, BCAT1, COCH, HTR2B, FAT1, IGSF3, MMP12, SLC12A1, TMC5, GTSF1, REG1A, GAGE1, COL4A5, SCN1A, TINAG, LOC284889, CES1P1, STK39, CLGN, CTHRC1, PAGE1, SBSPON, LRFN5, LY96, S100P, MUC13, TCEAL2, FABP5, LOC284561, TENM2, GJA1, C12orf75, TMEM45B, CCL20, GPX2, COL1A2 |
|  | Downregulated genes | LIFR, ERLIN1, CPEB3, KDM8, CLEC1B, LINC01093, SORBS1, RCAN1, HAMP, IL1RAP, GADD45B, HAO2, MFSD2A, MPC1, GPR128, ID2, OIT3, ASS1, FCN2, LARP1B, FCN3, SLC25A47, CXCL14, TTC36, PRG4, FAM65C, AKR1D1, ESR1, HHIP, ZG16, TUBE1, GRAMD1C, PITPNM3, GCH1, FERMT2, PLSCR4, GNPNAT1, IDO2, AGTR1, KLHL15, MT1F, CLEC4G, RSPO3, INTS6, SIK1, CDC37L1, STAB2, GLS2, CRHBP, PTH1R, MASP1, KLF6, CYP2B6, PSAT1, MT1E, FBP1, KMO, JUN, MT2A, FAM13A, EPB41L4B, PDLIM5, SOCS2, ETS2, HGF, CYP4V2, ZGPAT, FREM2, IL18R1, MT1X, PTP4A1, NAAA, MT1H, SPP2, SLC22A1, APBA1, NCOR1, MAP2K1, ECM1, AADAT, CXCL12, MT1HL1, PLGLB2, HAL, ETFDH, SKAP1, ANGPTL6, TMEM27, TDO2, HRSP12, GPR182, TMEM56, APOF, C21orf91, CLEC4M, C8orf4, SLC25A20, PPAP2B, THBS1, HOOK1, NAT2, LPIN2, ALDOB, LCAT, PPP2R1B, MT1M, MUT, BDH2, RDH16, AQP3, PCK1, CYP26A1, RNF125, ERRFI1, MT1G, PTS, N4BP2L1, CYP4A11, RBMS3, SH3YL1, TNFRSF10D, BCO2, CYP39A1, PLAC8, CNGA1, NPY1R, EGR1, CLN8, SYNE1, GAREM, FABP1, SPIDR, PDE7B, ZFP36, CD5L, GHR, MCC, LY6E, PPP1R1A, SLC7A2, AMOTL2, GOT2, SLC38A2, JUNB, SDS, MAN1C1, SLC19A3, ACADL, EPB41L4A, TBX15, RND3, ATF5, FOS, DACH1, PANK1, ANXA10, VNN1, COLEC10, CA2, C1RL, GLT1D1, IGF1, FBXO21, C1orf168, CYP2C18, MARC1, GOT1, CDH1, YPEL2, CYP2A6, GNMT, CYFIP2, SPATA6L, DHODH, CEBPD, PAIP2B, RIPK4, THRSP, EPHA2, GYS2, ID4, CHST9, RAB27A, LOC102723864, CNTN3, OGDHL, ACSL1, ABHD2, GPRASP1, KLF11, CDHR2, CIDEB, RP11-96D1.11, CXCL2, ZBTB21, HGFAC, CNDP1, CYR61, DUSP10, ARRB1, STOM, C9, B4GALT1, A2M, PRRG4, C3P1, ART4, ADK, PXDC1, DBH, EPB41L5, PCDH9, IGFBP3, ARHGEF26, FAM149A, ACOT12, KLRF1, DPF3, TMEM30B, CSRNP1, COLEC11, DUSP1, TENM1, HSD17B2, ALLC, KCNN2, CETP, DUSP5, IL6ST, FEZ1, FAM134B, MARCO, BLNK, IGFALS, OLFML3, PLIN2, ACAA1, EHD3, PGLYRP2, TCF21, SRD5A1, FXYD1, DMGDH, FTCD, NRG1, CYP2C8, C8B, LPA, DSEL, CNTN4, COX7B, FOXO1, EVC, DCN, ID1, FGFR2, SORL1, ANGPTL1, CYP1A2, DNASE1L3, IL4R, HMGCS2, CFP, FNIP2, AVPR1A, SLCO4C1, BMPER, SPRYD4, GPR125, GATA6, STARD5, GK, MYOM2, ADH4, NAV2, FMO2, MYO10, SATB1, CYP2J2, PLD1, ANKRD55, MAT1A, AK025288, NFKBIZ, RCL1, C7, SPRY2, CCBE1, GREM2, SLCO1B3, KRTCAP3, KBTBD11, ATP11C, NAPSB, TIAM1, DNM3OS, SERPINA5, ZC2HC1C, PRKAG2, ARID4A, LDLRAD4, CDA, NDRG2, UAP1, SLCO1B1, SLC4A4, HAND2-AS1, SLC27A2, PPARGC1A, GLYAT, ACMSD, SLC22A7, ADRA1A, GBA3, DAPK1, ELMSAN1, ACSM3, RASGEF1B, TMPRSS2, FGA, CPN1, PALM2, GLDC, APOA5, CYP2C9, ADAMTSL3, ASPA, IYD, MGLL, CBS, SUCLG2, SLC10A1, CLDN1, SLC39A5, TCTEX1D1, PIK3C2G, KAZN, SLC25A18, ITGB8, INPP1, CYP2C19, VIPR1, PHGDH, NFIL3, TTC39B, HPGD, GPM6A, ITGA9, RAPGEF5, SMAD9, ALDH6A1, SLC25A15, ANK3, IL13RA2, AKR7A3, MOGAT2, ATOH8, PTGS2, COL6A6, SHBG, MCTP2, ATF3, ABHD5, AJUBA, XDH, FOXA3, SRD5A2, GPR126, SRPX, PZP, TRIB1, PPM1A, LIPG, TAT, DEPDC7, LDLR, CTH, SPATA18, AGPAT9, GLIS3, FETUB, LSR, MCL1, ACADSB, ASL, EXOC3L4, CYP2A7, ANGPTL3, ZFP3, FRMD4A, KLKB1, GABARAPL1, CP, HRG, MRO, RNF165, FAM110C, WDR72, SLC51A, GPD1, AKAP12, CD14, MRGPRF, FOSB, MYH10, GSTZ1, MBL2, FRMD6, SLC2A9, SLC16A2, OAT, CD1D, SLC41A2, IER2, CHST4, ADAMTS13, CCDC71L, QDPR, ABLIM3, BCHE, GADD45A, PEMT, SULT1A2, FOXF1, GCDH, BMP5, HAO1, CYP4F2, AMDHD1, LRRN3, C6, LINC01146, CTB-167B5.2, MST1L, CROT, STAT4, NR4A2, BGN, PDK4, CYP2B7P, ST6GAL2, STEAP4, SERPINA4, ANO1, TDRD6, CCDC68, BTG2, EGR2, GBP1, TGFA, SLC25A27, PAPSS2, FDX1, CNTLN, NAMPT, TACSTD2, IRF8, STAT3, SLC38A4, RNA45S5, TSPAN12, IL18RAP, TSPYL5, ANGPTL4, FAM150B, ST6GAL1, SLC25A25, ADAMTSL2, RAPH1, BHMT, G6PC, CLIC6, EPCAM, ALAS1, F11, UNC93A, CYP3A43, ELF3, ENO3, SAMD5, TSLP, TMEM45A, SERPINB9, AFM, DHRS1, ADAMTS1, KIAA0922, SLC20A1, ETNPPL, IGJ, SEMA6D, CAND2, SGK1, TJP2, ACACB, P4HA1, SULT1A1, ABI3BP, FAT4, PAMR1, RDH12, DIRAS3, MASP2, RNF180, ADCY1, IPCEF1, AZGP1, FNDC5, BBOX1, C11orf96, ZFPM2, SHROOM2, PHYH, RAB25, LINC01018, LOC200772, FAM9B, ZNF667-AS1, TTR, PRR18, ESRP1, WWC1, MBNL2, PCOLCE, PON3, TMEM178A, TMEM154, RP11-469M7.1, CFHR3, IGFBP1, ZFP1, FAH, KLF10, HPX, GJB2, PFKFB3, ARG1, TMEM200C, SCARA5, HSPA4L, C8A, INSIG1, PROZ, SYNPO2, CFTR, FOLH1B, LURAP1L, HES1, LOC101928505, DNALI1, PELI2, ACAA2, GPR180, LTBP1, SLC23A2, PLCG2, SLC1A1, CCDC3, BHLHE40, TRIM22, TLR3, CHST7, GIPC2, ABCA8, INMT, SPIRE1, SDC4, PXMP2, ALDH8A1, ADH1A, STEAP3, PTGIS, DLL1, LONRF2, ITIH4, CD69, GSTA3, EPHX2, SLC44A1, APOA1, F9, AGXT, GK3P, HMOX1, ASPN, FHL1, CYP3A7-CYP3AP1, PRKAR2B, KYNU, GDA, LHX2, KCNMA1, BHLHE22, GUCY1A3, CPED1, SLC22A25, PDGFRA, TEK, APP, EPHA3, CTNNA3, FLRT3, C1R, PCDH18, CBR4, MEST, CLRN3, SERPINA6, INHBE, GABRP, ADH1C, KCNJ16, SOCS3, FAXDC2, IGHM, RGN, SPINT2, SLC13A5, A1BG, KCND3, LOC100129447, SVEP1, GSTA1, MYC, RFTN1, GRAMD4, ORM1, SC5D, CFHR4, XBP1, LOC286114, IGKC, DPT, ARRDC4, COBL, SLC9B2, TRPM8, PLG, NRBF2, DPYD, SOX6, LUM, CLDN10, DUSP2, MXRA5, RAB20, KIAA1671, REEP6, HSD17B6, LRRC31, UGP2, ATP2B2, CYP3A5, SLC17A1, RHOB, STS, SCD5, CDH19, IL33, SDSL, AUTS2, HABP2, BASP1, DCPS, PPP1R3B, MS4A6A, LEPREL1, GEM, ARMCX3, LOC100505985, FXYD2, BEX1, TFPI2, SERPINA7, IGLC1, FOXP2, RBP4, PHLDA1, PLSCR1, DEFB1, GZMK, F3, CLU, SPAG1, TRIM15, GRHL1, EIF4E3, HPD, RGS4, AGXT2, NALCN, SEC14L4, KLRB1, SULF2, CRISPLD2, FZD7, RECK, MUM1L1, ANG, MRC1, IGLV1-44, SAT1, SLPI, RP11-138A9.1, DOCK8, TTC39C, FCRL3, GPRIN3, CRP, GAS1, F13B, CLSTN2, LOC286087, TIMD4, GABRB3, GPR171, FCGR2B, DSE, GPC6, LGSN, SERPINE1, F2RL1, DPYS, OLFM1, FZD1, PRNP, USP2, VNN3, SLC15A1, ADORA3, MAP3K5, KLF4, GDF15, PNP, PRELP, SCARNA17, BEX4, LYVE1, PWAR6, USP9Y, FLJ22763, ANXA3, TFR2, PLEKHH2, SPG20, FGL1, KLF9, FILIP1, SEMA5A, HP, GADD45G, APOM, EPHB1, CD9, CR1, FBLN5, LRG1, PON1, ADH1B, CDKN1C, CTGF, KCNS3, ADAMTS2, MS4A7, IGLL3P, WASF3, NR1I3, MMRN1, CYP3A4, LEAP2, SLC27A5, HBB, SYT17, RGS1, KNG1, KANK4, SERPINF2, FIGN, EDNRB, CD274, GPLD1, PDE1A, BCL2, ADH6, ACSM5, PKHD1, RP11-373D23.2, CDO1, CFD, LOC149703, CD8A, CCL2, LECT2, AMIGO2, LOC101928635, RASD1, CYP2E1, AHSG, NNMT, IL7R, TMC4, ITK, IFITM1, MS4A1, CPS1, UPB1, SCN7A, RGS2, APOC3, ID3, SERPINA3, ERICH5, PDZRN3, KRT7, CCL5, NR5A2, P2RY13, TAGAP, S1PR3, COL14A1, IKZF1, ADAMTS17, SYTL3, FST, PTPRC, UPP2, FILIP1L, EPB41L3, RP11-1151B14.3, CYTIP, AXL, CH25H, MAL2, THBD, SLC3A1, ARL4C, CXCL6, FAM26F, EVI2B, BHMT2, CHRDL1, SULT2A1, CCL19, SLC2A3, LOC100507389, MPEG1, FGL2, SLC2A2, S100A8, COL3A1, THBS2, DSG2, ASCL1, SAMSN1, PEG3, PTGER4, CYP8B1 |
| GSE98383 | Upregulated genes | HEATR2, SLC7A11, SLC38A6, CENPW, XPOT, ZFP41, ZIC2, HOXA13, LOC344887, HMMR, CCDC34, RRAGD, ZSWIM5, CAP2, ANLN, DNAH12, PSPH, RNF187, NUP155, TMEM74, E2F7, CENPU, TIMELESS, GARS, CCT6A, CDKN3, SRPK2, DTYMK, GPR158, PHTF1, MSTO1, FAM83D, GEMIN6, OIP5, HOMER1, BAG2, ZNF692, MNS1, CCNB1, TINAG, LOC101928000, DNAJB6, FDPS, NRAS, EME1, EIF3B, EZH2, MELK, TIPIN, TOMM40L, NDUFB9, UTP15, MAP2, PRIM1, FBXL18, LSM4, KIF20A, MND1, STEAP2, PSMG3, CCNA2, KIF15, SPSB2, DNAJC6, FEN1, CCDC93, ROBO1, STMN1, NCAPG, APOBEC3B, TTK, WDR12, TK1, TTLL7, BIRC5, TMEM64, ABCF2, DIAPH3, RP5-1136G13.2, COPS6, CHCHD3, ILDR2, PBK, NCAPG2, H2AFZ, XPO4, CENPH, E2F8, UBE2T, NEK2, TRIM37, DEPDC1, NAA20, AP000525.9, RRM2, SQSTM1, GINS1, DR1, ASPM, ANP32E, DCAF13, ZNF165, MCM4, LL22NC03-N14H11.1, HJURP, SPDL1, FLAD1, SESN3, BUB1, SSR1, LAPTM4B, FIGNL1, ZWINT, HMGCS1, AKR1C1, RP1-228H13.5, TRIM24, TCEB1, CKAP2, CENPM, COA1, TAB3, POLR3G, SESTD1, SQLE, TBCE, DTL, PIEZO2, GBAP1, C1orf85, CKAP4, TBC1D16, CNKSR2, DLGAP5, CDKN2C, CCNE2, DUXAP10, KPNA3, IMMP1L, SAC3D1, PRC1, KIF14, FANCI, PPAT, HTATIP2, LOC730101, HEXA, OSGIN1, TOP2A, TKT, KCTD6, STEAP1, DLAT, CDC7, LOC101928545, WASF1, KPNA2, CTB-92J24.2, TUBG1, UBE2C, RP11-932O9.10, SLC40A1, HLTF, TCF19, KIAA0895, NSMCE2, CDK5, ESPL1, AKR1C3, CNIH4, ENAH, TMEM106C, TRIM71, TRIM16, STIL, BRIP1, SLC4A2, RFC4, FARP1, TPD52, MSH2, CDC25C, SEC61A2, PTK2, KIF4A, CDCA3, DNAH14, DSCC1, TRIP13, NUP205, LOC389834, NDC80, RFC3, TGM3, OGG1, RAD51, AP1M2, FTH1, CDK1, MKI67, MCM10, ECT2, MCM8, RAD54L, C8orf33, ZWILCH, LRRC45, GGCT, CCNB2, GLA, MZT1, CENPF, TYW3, ABCB6, RALA, RAB29, EFCAB2, SGOL2, SIX1, DNAJC3-AS1, NUPR1, PAFAH1B3, NEU1, C7orf49, SSBP1, DANCR, ARPC1A, CD109, SPX, MCM7, RAD51AP1, SKIDA1, FAM83H, ZNF252P, CEP152, COCH, ATG10, PTTG1, FUCA2, NUSAP1, ACACA, PTDSS1, TXNRD1, PRPSAP1, PARPBP, NPM1, GNAL, NQO1, SYT1, DONSON, KIF23, RNASEH2A, MCUR1, ASF1A, MTFR2, MAD2L1, CDKN2A, NAA50, MPP6, RACGAP1, EML6, LRIF1, CACYBP, PIR, MCM3, SMKR1, TMEM97, OTUD6B, TES, NUDT1, MIS18A, TBC1D7, GNPDA1, BUB1B, RP11-295G20.2, CDC20, SLC35G2, SSX2IP, KLHL13, MAP3K9, ZBED6CL, CAV2, CHEK1, FOXM1, ONECUT2, SMEK2, GCNT3, POLQ, KIF2C, WNT5A, DDIAS, PDSS1, DUT, HS2ST1, GREM1, B3GNT5, ZSCAN9, FAM84B, NUF2, METTL21A, TMEM14A, RP11-29H23.4, FNBP1L, CCDC150, CDCA8, NCAPH, SATB2, CIART, PTGFR, TLCD1, CENPI, GNAI1, DBF4, RAD54B, APTR, CLIP4, NDC1, MPP7, PLK4, HMGCR, SPC25, AIMP2, EIF4EBP1, AGFG1, C4orf46, STRA13, ZNF697, ASPH, CDC45, CDCA5, SCLY, CCNYL1, COL28A1, ERC2, ZSCAN31, LSS, STC2, SMC4, PTGR1, DPH3, NREP, DLEU1, FRY, NOX4, CBX3, MS4A8, CDCA7L, ME1, PHEX, GAS2L3, CDC6, NME1, CLDN12, ACSM1, TMEM144, CHEK2, ORC6, PDCD2, PPP4R4, G6PD, OTUD6B-AS1, ZNF57, DDC, DPY19L1, TRIM45, C12orf75, FN1, RBM24, SNX7, UGDH, BRCA1, MCM2, GPSM2, CENPK, KIAA0101, LOC401068, GPC3, HILPDA, DDIT3, CENPE, EXO1, IL17RB, KIF18B, RIT1, PACSIN2, OSR2, PDZK1IP1, SARS, RUSC1-AS1, TTC22, RRAGC, DENND2C, NEIL3, TFAP2A, HKDC1, CSTF3, CEP55, TRIB3, KIF11, AP1S1, FAM133A, ALPK3, CEP85, CENPJ, STXBP6, DEPDC1B, PPM1E, PMFBP1, LPL, COL24A1, PF4V1, AURKA, DPH6, RRAS2, LOC100506922, SPA17, ATP6V1C1, TYMS, MCM6, SKP2, POLE2, MANEAL, HELLS, RECQL5, PRMT6, IGSF3, SPAG5, KHDRBS3, CDC25B, GMNN, PRR11, FANCD2, BAMBI, FLVCR1, IGF2BP3, FHIT, CKS2, XK, TPX2, RNF213, STX3, CDKN2B, SASS6, ARHGEF37, SRXN1, UGT1A6, CNTNAP4, LOC100507316, SULT1E1, SOAT2, CNPY2, TET1, ZNF174, H2AFX, VSIG10L, SPINK1, PLA1A, LOC728392, SSX1, PKIB, LOC100289098, IL17D, MTHFD2L, LINC00348, LAMTOR4, DCAF4L2, KIFC2, LINC00622, TACC3, VSNL1, MAP2K6, IL1R2, UHRF1, SPHK1, COQ10A, GPX7, SLC44A5, SIX4, KIAA1841, SMPX, CDC20B, SLC16A10, KLB, LOC100505851, UNK, CHML, LOC102723847, CCNE1, MRPS12, KIF18A, SPP1, SAP30, TBC1D30, TSPAN12, FAM124B, LINC01419, SULF1, SMC2, INSC, GP2, EID3, FAM184A, ETV1, CR936796, LOC101928076, ENPP6, SIPA1L2, AFAP1-AS1, CERS6, PCOLCE2, LYPD8, EGF, RAB38, PTGR2, STAU2, CSTA, HOXA3, MATR3, AFP, C2CD4A, TFRC, RBM20, TRIM6, SV2B, JAKMIP3, SLC6A13, SLC12A8, SLC30A10, DNER, BCL2L10, MAGEC2, SPOCK1, KRT12, AKR1B10, SPATA41, ST8SIA6-AS1, MBD1, ZBTB41, TRHDE, MACROD2, NRCAM, LOC284561, FGF13, LOC100507477, DACH2, COG3, RNF157, ERICH5, GRP, TTC39A, CYP17A1, XYLB, COL5A3, COX7B2, VAT1L, UNC5D, CACNA1E, EYA1, NR0B1, RPL22L1, CYP7A1, DFNA5, CMBL, HINT3, GUCY2C, ARHGAP36, SULT1C2, LINC00942, SLC28A2, RP11-847H18.2, ZNF311, ISX, UPK3A, ASRGL1, LRP4, CDH12, PAGE4, LIN28B, LOC101928820, TEX11, TMEM163, DAB1, RYR3, SPRR3, COL15A1, HAGLR, POPDC3, CRNDE, SLC2A5, SCGN, TM4SF20, FAM9B |
|  | Downregulated genes | CXCL14, HHIP, CD5L, MASP1, LYVE1, TMPRSS2, TMEM154, ECM1, RSPO3, C8orf4, CCBE1, ADAMTS13, GADD45B, CDH19, MARCO, PLSCR4, TTC36, LY6E, CSRNP1, PTH1R, DLC1, FAM65C, ANGPTL6, PEG3, CTB-167B5.2, SIK1, GPM6A, GALNT16, FLJ38379, C14orf105, LCAT, IGFBP3, P4HTM, OIT3, DNASE1L3, CXCL12, DBH, TCF21, DDB2, INMT, TIMD4, RND3, MT1E, CFP, ETS2, STEAP4, GFOD1, LGALS3BP, LIFR, DACH1, SRPX, CYGB, MAN1C1, EPCAM, COLEC10, ANKRD18A, KBTBD11, CETP, IFITM1, MT2A, IGLC1, GABARAPL1, CCDC68, ADAMTSL2, DUSP1, ERBB2, CR1, SLC25A25, TBXA2R, PDE2A, MFSD2A, CLEC1B, C7, ZNF667-AS1, STAB2, APOA5, ADORA3, TMEM47, PTPRN2, OAT, MYO10, PPAPDC1A, CRHBP, LOC200772, CLEC4G, LOC285812, CCL19, HGF, GNA14, LHX2, KAZN, CFD, CXCL2, DPT, PLCXD3, TACSTD2, EPHA2, ATOH8, GK, PPP1R3B, DCN, OAS2, NME5, MZB1, COX7A1, ANTXR2, AGTR1, SLC23A2, OLFML3, HSD17B2, RBMS3, FAM150B, TGOLN2, TAF4B, MEIS3P1, RPH3AL, GPR182, RCAN1, PDGFRA, NTF3, VIPR1, PKHD1, SLCO1B3, SIGLEC7, OLFM1, IGLL3P, CLDN10, MT1X, LDB2, FCN3, GRAMD4, SFRP1, BST2, SVEP1, SYNE1, HENMT1, COLEC11, ZBTB21, CST7, KLF11, EXOC3L4, EGR1, HAND2-AS1, PRDM1, RNF125, GGT5, ASGR2, LEPREL1, NOSTRIN, BEX1, MS4A1, SLC9A9, ANKRD36BP2, MFAP4, FCRL5, FYN, DNM3OS, SMIM24, TSPYL5, IRF8, TNFRSF17, NNMT, BHLHE22, PLA2R1, ITGA9, THBD, CCND1, SDC3, GCH1, TMED3, TEK, PAMR1, CYP2E1, IGJ, KLRF1, FOXO1, TNFRSF1B, MS4A7, GSN, MBL2, GAS1, EDNRB, SLC38A2, CHST4, LTBP4, FCN2, ADAMTS1, ZFP82, CD14, SMAD6, FBLN5, PGLYRP2, FOLR2, BMP5, COL4A3, PTGIS, SYTL5, CLSTN2, GPR128, IGLL5, P2RY12, ZFP36, DUSP2, CNTN4, KIAA0125, TTC39B, VMO1, HS3ST3B1, RGS4, CCDC3, ROBO2, CPED1, GUSBP11, ANGPTL1, BAI3, NDN, CFTR, IFI6, C11orf96, SYNPO2, BACE2, IL2RB, SELP, IL4R, C1R, TNFAIP2, EFCAB4B, SCNN1A, NR4A2, GHR, PTGDS, TMOD1, GRK5, PRKCB, CYR61, RNF150, CDKN1C, P2RY13, AMICA1, FCRL3, CD163, CLIC6, TSPAN7, SPIDR, ENG, IGLV1-44, DOCK5, SYTL3, GPC4, TVP23B, IRF7, SCN7A, NFASC, EPB41L4B, CRISPLD2, EPHA3, TXNIP, KMO, MT1HL1, ZNF471, IGHM, LILRA2, CNRIP1, PLAC8, IRF9, LOC101927653, BTG2, FXYD6, MX1, SOCS2, MS4A6A, TMEM100, ADH1B, FAM83F, COL4A4, HIST1H2BC, LY9, FAM19A5, EVC, GATA6, RHOH, PITPNM3, ATP8B4, SLC25A47, IL33, LAMA2, LONRF1, C1RL, ANKS1A, ZFPM2, TMEM86B, FOS, IGLJ3, SDPR, IGKC, NEURL1B, PLA2G2A, LOC100507311, EFEMP2, ASPA, RNF135, ISG15, PIP5K1B, WWC1, ZNF415, EMILIN1, PTPRB, ADAMTS2, KCNS3, GIMAP1, PPAP2C, AOC3, KCNJ10, NXPE3, ITGBL1, TCTEX1D1, CKMT2, PPP2R5C, CD27, AMIGO2, THBS1, CCDC71L, ENDOD1, PYROXD2, BCL11B, CYP4A11, SPATA18, PELI2, FHL2, NCAM1, PLA2G4A, IGK, ESRP1, ARHGAP25, CYFIP2, ZEB2, RUNX3, SYT9, BCO2, NAAA, RASD1, PNMAL1, PPP1R16B, PDE1A, ID3, SPP2, PHLDA1, FLJ32255, SCD5, RBP1, SYTL2, TIMP3, IGF1, MRC1, LAMC3, PDGFD, DDX26B, SLC25A36, HBB, ITGB2-AS1, A2M, FMO2, ETS1, LRG1, EVA1C, PCK1, ZNF160, CXCL16, CACNA2D1, KDM8, NPY1R, NFATC1, DDR2, MPEG1, GPRASP1, ADAMTS9-AS2, CD69, TMC8, KRTCAP3, BEX4, GMFG, CYP2C9, EIF5, FPR1, BASP1, C8B, TMEM204, GALNT3, NAPSB, PARP8, GPR56, TMEM178A, CCL4, PNOC, ARRDC4, PIK3R5, ASGR1, WFDC1, RP11-38P22.2, NTN4, SGCD, TLR4, NEXN, PEG3-AS1, GSTM5, VSIG4, SLC1A2, MAP9, BGN, OAS1, XAF1, RAP1GAP2, TCEAL3, DKK3, ITGA1, IFITM10, SEC14L4, ZNF83, PRF1, AQP1, RBMS1, PBLD, CCR2, TRIM22, AQP3, FAM46C, TGFBR3, MT1F, SERPINA7, FGB, ID4, SLC28A3, FGFR2, GIMAP6, CFH, KIAA1324L, PTGS2, BMS1P20, ZNF518B, TGM2, CD36, QSOX1, CLMP, PTGDR, C1QTNF7, SH3BP5, CDHR2, TRG-AS1, LEPR, CYP2B6, AKAP12, FOXF1, DACT1, SLIT2, IL1RL1, IL10RA, ALAS1, NBEA, KLRB1, ANK3, MRO, CCL5, IFI27, GNMT, PCDH18, FLI1, CD48, ANKRD36B, HLF, FAIM3, CXCR4, DLL1, CECR1, NDRG2, KLF2, IL1RN, IL18R1, NALCN, FOXQ1, ANKRD36, RCL1, LECT2, C16orf54, SELL, LAT, ST6GAL2, F3, CEBPD, MT1H, IER2, LINC01093, ADH4, LAX1, IL13RA2, CCR7, CDKN1A, SLC44A2, RGN, TIMP2, SLC44A1, PDZRN4, PDE7B, GABRP, TNFRSF10D, RGS9, RECK, CCDC88C, VAV2, STK4, PEAR1, LBH, ITK, C8A, RAI2, SCML4, IDNK, LOC100505570, PVRIG, SLC13A5, TENM1, CSF2RB, LILRB2, ADCY1, STX11, IGHD, GPRIN3, UBASH3A, PRKCQ-AS1, FRMD4A, DHODH, HCLS1, CMPK2, PRICKLE2, DAPK1, KLF10, ZNF137P, FAM65B, TRBC1, EMR1, TGFB1I1, NSUN6, PRKAR2B, RNASE4, LGR6, AKNA, MAMDC2, C1orf162, PRKG1, TRAC, GNG2, AXL, MXRA5, WIPF1, TCF4, RCAN2, ORM1, PIK3CD, PTN, RP11-642D21.1, C1QB, CFB, FOSB, CTH, TMC4, IRAK3, IP6K3, DSE, STEAP3, TUBE1, EVI2B, EMB, GADD45G, ACKR1, OLFML1, TRIB1, BACH2, GZMK, AIM2, KRT19, KANK4, SLC1A7, CSF1R, RUNX1T1, IL18RAP, JUNB, TLCD2, CLEC4M, HSPA2, CD38, CD55, HPX, LUM, PEMT, FEZ1, CYTIP, SLAMF7, TAGAP, SERPINE1, C1QA, HOTS, DOCK2, CYSLTR1, SPON1, DUSP5, SLC22A7, PIWIL4, EOMES, NAMPT, RAB25, RASSF2, KLF4, SLC51A, CYBRD1, CCND3, EML1, PTPRS, NLRC5, SLC16A14, CH25H, SLC8A1, TMEM200C, SERPINA1, TRAF3IP3, C8G, CLDN11, BLK, LOC100289058, N4BP2L1, RP11-1151B14.3, SELPLG, TMEM56, IFI16, GPT2, TMEM45A, PLEK2, PGR, ASPN, ESR1, IGFBP7, PTPLAD2, FRZB, TMEM173, SFMBT2, IL7R, WFDC2, C4BPA, ID1, HGFAC, SLA, MCOLN2, C1QC, PTPRC, TNFSF11, ARGLU1, SLC2A9, CD8A, SPRY1, PDZRN3, IFIT1, CCL21, AGPAT2, CELF2, PLAGL1, SSPN, FABP4, FGA, TPBG, TNFSF8, SERPINB9, SLC31A2, NRXN3, NR2F1, PIK3CG, PRR18, NLRC3, ZC3HAV1L, GBP5, CA5A, GK3P, PRICKLE1, FNDC5, ITGB8, IGFBP6, RASGRP1, GLIPR1, FGL2, IPCEF1, RNF165, FGD3, SRGN, CAMK4, ANO1, RAC2, JAK3, ALLC, GPR171, ADH1A, THEMIS2, GEM, RORA, GLT8D2, ECM2, AEBP1, INHBE, SMAD7, S1PR3, FGF1, CRIM1, CLEC7A, PBX4, GUCY1A3, CLEC11A, NCKAP1L, COL4A5, C1orf116, RORB, CTGF, C2orf40, RAB31, ABCG5, GOT1, AFM, TAL1, CSGALNACT1, STOM, FILIP1L, FABP1, ITIH3, FBP1, DGAT2, P2RY14, ADRBK2, CDC42EP3, CP, KLHL6, PRKCH, TNFSF13B, EPB41L3, SERPINF2, ADRA2A, ARHGDIB, ADCY7, BCL2, KCNAB1, SAMSN1, H1FX, HLA-DOA, GVINP1, ITIH4, CD53, P2RY8, CALD1, TLR8, ZNF468, GZMB, SH3RF3, RCSD1, GZMA, CHSY1, ANTXR1, LCK, INPP4B, LOXL4, TRAT1, NAV2, P2RX5, FAM3B, ACSL5, SLC2A10, SELM, FAM26F, TCEA2, A1BG, VIM, C9, GREM2, MYL9, MEG3, UBXN10, RP11-747H7.3, CLSTN1, SMIM3, ZNF677, GNB4, NR4A3, COL6A1, TAGLN, SGK1, PTPRH, RP11-96D1.11, TGFA, RGS2, ANXA3, ZSCAN18, GPX3, EGR3, PSMB8-AS1, NTS, KCND3, EFEMP1, TMEM71, CPA3, ARHGAP30, COL13A1, FMOD, MTHFD1, PAX5, LINC00342, FAM110C, LHFP, APOF, MX2, LEAP2, GPR65, HAMP, MSR1, BANK1, SERINC2, PREX1, SH2D1A, MYOM1, CLRN3, KCTD12, WASF3, KCNJ16, S100A11, DPYSL3, LTB, ITGA4, LDHB, LINC00924, CD97, ALDH1A3, DOCK11, MAGEH1, CXCL5, COTL1, KAL1, CD52, SMOC2, MYH4, PTGER4, FMO3, CHST11, ADH1C, LXN, HPD, TDRP, MT1G, AREG, ITGB2, S100A8, CIDEB, VCAM1, ABCA8, AMPD1, TYROBP, GPR64, LPA, PNMA2, PPP1R14A, EVL, TMPRSS3, PMP22, ATF5, ANG, CRTAM, MT1M, ARL4C, SH3YL1, DLGAP1-AS3, PLG, HCK, EVI2A, RASSF9, CTLA4, CYP2C8, KLF7, CYBA, CD2, LTBP1, PDP1, ZNF559, SNX20, PROCR, THEMIS, ZKSCAN7, C6, MASP2, RDH16, ZNF320, KRT7, COL14A1, SLC22A17, PLEKHH2, MOXD1, RP11-305O6.3, CCND2, LY75, CYP4V2, SOCS3, ARID5B, ALDOB, TRPM8, TSC22D3, MOGAT2, CYP1A2, CXCR6, MTHFD2, TAT, MMP2, DACT2, CYP4F2, METRNL, LPAR1, ANXA13, APOA1, DLK1, FAM43A, F11, CCL2, GYS2, MFI2, EGR2, HOXB3, TESC, RNASE6, MYRF, PLEK, BMPR1B, DNAJC12, NAT2, PLAT, RGS18, ZNF331, AQP9, KIAA0226L, CORO1A, HAL, GRAMD1C, SERHL2, SLC7A2, HLA-DPB1, CYP8B1, OSBPL10, OGFRL1, IFI44, SLC25A15, IFI44L, CXCL9, VLDLR, PLTP, TRPC1, MYOF, IQGAP1, SLC6A6, ASS1, ALOX5, IGSF6, ARMCX1, WISP1, THRSP, STON1, SDS, GABBR2, A2M-AS1, ACSM5, FPR3, PROM1, WT1, TMEM200A, G6PC, HEPH, PMEPA1, RUNX2, DCDC2, RGS1, UCP2, ASL, CPN1, SMAD9, MSRB3, CPS1, ACTA2, ABCB4, MGP, F9, STMN2, SLC17A2, SPARC, ADSSL1, ACSM3, CPEB3, TDO2, MYADM, HTR2B, COL6A3, DYNC2H1, LIMCH1, SNORD114-3, PFKFB3, ZG16, SYT13, THBS2, ENPP5, CDH1, OSMR, SERPINE2, LAPTM5, CLDN2, EPHA4, AHSG, CFHR3 |

,
