# Supplementary material for: Microarray Data Mining and Preliminary Bioinformatics Analysis of Hepatitis D Virus-Associated Hepatocellular Carcinoma
Source: Biomed Res Int. 2021 Jan 30;2021:1093702. doi: 10.1155/2021/1093702 (PMC7867452; doi:10.1155/2021/1093702)
Supplement: Supplementary Materials — Table S1: DEGs from microarray datasets GSE55092 and GSE98383. Table S2: 948 DEGs related to HDV-associated HCC including 373 upregulated and 582 downregulated genes. Table S3: the five modules and contents obtained by WGCNA. [file 1093702.f1.zip › Table S2.docx]

| Table S2. 948 DEGs closely related to HDV-associated HCC including 373 upregulated genes and 582 downregulated genes | |
| --- | --- |
| DEGs | Gene name |
| Upregulated  genes | HEATR2, XPOT, RNF187, NUP155, TMEM74, TIMELESS, GARS, CCT6A, SRPK2, DTYMK, PHTF1, MSTO1, GEMIN6, OIP5, HOMER1, BAG2, ZNF692, MNS1, DNAJB6, FDPS, NRAS, EIF3B, TIPIN, NDUFB9, UTP15, FBXL18, LSM4, MND1, PSMG3, CCDC93, WDR12, TTLL7, ABCF2, DIAPH3, RP5-1136G13.2, COPS6, CHCHD3, NCAPG2, H2AFZ, XPO4, CENPH, UBE2T, TRIM37, NAA20, SQSTM1, DR1, ANP32E, DCAF13, ZNF165, MCM4, FLAD1, SESN3, SSR1, LAPTM4B, HMGCS1, AKR1C1, RP1-228H13.5, TRIM24, TCEB1, COA1, POLR3G, SESTD1, SQLE, KPNA3, IMMP1L, PPAT, LOC730101, OSGIN1, KCTD6, DLAT, LOC101928545, WASF1, KPNA2, RP11-932O9.10, SLC40A1, HLTF, KIAA0895, NSMCE2, ESPL1, CNIH4, ENAH, SLC4A2, FARP1, TPD52, SEC61A2, PTK2, DNAH14, NUP205, RFC3, TGM3, OGG1, RAD51, AP1M2, FTH1, MCM10, C8orf33, ZWILCH, GGCT, GLA, MZT1, TYW3, RALA, RAB29, SIX1, DNAJC3-AS1, NUPR1, PAFAH1B3, NEU1, C7orf49, SSBP1, DANCR, ARPC1A, MCM7, SKIDA1, FAM83H, ZNF252P, ATG10, FUCA2, ACACA, PTDSS1, PRPSAP1, PARPBP, NPM1, SYT1, DONSON, KIF23, RNASEH2A, MCUR1, ASF1A, NAA50, MPP6, LRIF1, CACYBP, SMKR1, TMEM97, OTUD6B, TES, NUDT1, MIS18A, TBC1D7, GNPDA1, SLC35G2, SSX2IP, KLHL13, MAP3K9, ZBED6CL, CAV2, ONECUT2, SMEK2, POLQ, DDIAS, PDSS1, GREM1, ZSCAN9, FAM84B, METTL21A, TMEM14A, RP11-29H23.4, FNBP1L, CCDC150, CDCA8, NCAPH, SATB2, CIART, PTGFR, TLCD1, CENPI, DBF4, RAD54B, APTR, CLIP4, NDC1, MPP7, PLK4, HMGCR, AIMP2, EIF4EBP1, AGFG1, C4orf46, ZNF697, ASPH, CDC45, CDCA5, SCLY, CCNYL1, COL28A1, ERC2, ZSCAN31, LSS, STC2, SMC4, DPH3, NREP, DLEU1, FRY, CBX3, MS4A8, CDCA7L, ME1, PHEX, GAS2L3, CDC6, NME1, CLDN12, ACSM1, TMEM144, CHEK2, ORC6, PDCD2, PPP4R4, OTUD6B-AS1, ZNF57, DDC, DPY19L1, FN1, SNX7, UGDH, HILPDA, DDIT3, CENPE, EXO1, IL17RB, RIT1, PDZK1IP1, SARS, RUSC1-AS1, TTC22, RRAGC, DENND2C, NEIL3, TFAP2A, HKDC1, CSTF3, TSPAN12TRIB3, AP1S1, ALPK3, CEP85, CENPJ, PPM1E, PMFBP1, PF4V1, DPH6, RRAS2, LOC100506922, SPA17, ATP6V1C1, TYMS, SKP2, POLE2, MANEAL, HELLS, RECQL5, PRMT6, SPAG5, KHDRBS3, CDC25B, GMNN, FHIT, CKS2, XK, RNF213, STX3, SASS6, ARHGEF37, UGT1A6, LOC100507316, SULT1E1, SOAT2, CNPY2, TET1, VSIG10L, PLA1A, PKIB, LOC100289098, IL17D, MTHFD2L, LAMTOR4, KIFC2, LINC00622, TACC3, VSNL1, MAP2K6, IL1R2, SPHK1, COQ10A, GPX7, SIX4, KIAA1841, CDC20B, SLC16A10, KLB, UNK, CHML, CCNE1, MRPS12, KIF18A, SAP30, TBC1D30, FAM124B, SULF1, INSC, GP2, FAM184A, ETV1, LOC101928076, ENPP6, SIPA1L2, AFAP1-AS1, CERS6, PCOLCE2, RAB38, PTGR2, CSTA, MATR3, AFP, C2CD4A, TFRC, RBM20, TRIM6, SV2B, JAKMIP3, SLC12A8, SLC30A10, DNER, BCL2L10, SPOCK1, KRT12, SPATA41, MBD1, ZBTB41, TRHDE, MACROD2, LOC100507477, DACH2, COG3, GRP, CYP17A1, XYLB, COL5A3, VAT1L, UNC5D, CACNA1E, EYA1, NR0B1, RPL22L1, CYP7A1, DFNA5, CMBL, HINT3, GUCY2C, ARHGAP36, LINC00942, SLC28A2, RP11-847H18.2, ZNF311, UPK3A, ASRGL1, LRP4, CDH12, LIN28B, LOC101928820, TEX11, TMEM163, DAB1, RYR3, SPRR3, HAGLR, POPDC3, SCGN, TM4SF20 |
| Downregulated  genes | DLC1, GALNT16, FLJ38379, C14orf105, P4HTM, DDB2, GFOD1, LGALS3BP, CYGB, ANKRD18A, ERBB2, TBXA2R, PDE2A, TMEM47, PTPRN2, PPAPDC1A, LOC285812, GNA14, PLCXD3, OAS2, NME5, MZB1, COX7A1, ANTXR2, TGOLN2, TAF4B, MEIS3P1, RPH3AL, NTF3, SIGLEC7, LDB2, SFRP1, BST2, HENMT1, CST7, PRDM1, GGT5, ASGR2, NOSTRIN, SLC9A9, ANKRD36BP2, MFAP4, FCRL5, FYN, SMIM24, TNFRSF17, PLA2R1, CCND1, SDC3, TMED3, TNFRSF1B, GSN, LTBP4, ZFP82, SMAD6, FOLR2, COL4A3, SYTL5, IGLL5, P2RY12, KIAA0125, VMO1, HS3ST3B1, ROBO2, GUSBP11, BAI3, NDN, IFI6, BACE2, IL2RB, SELP, TNFAIP2, EFCAB4B, SCNN1A, PTGDS, TMOD1, GRK5, PRKCB, RNF150, AMICA1, CD163, TSPAN7, ENG, DOCK5, GPC4, TVP23B, IRF7, NFASC, TXNIP, ZNF471, LILRA2, CNRIP1, IRF9, LOC101927653, FXYD6, MX1, TMEM100, FAM83F, COL4A4, HIST1H2BC, LY9, FAM19A5, RHOH, ATP8B4, LAMA2, LONRF1, ANKS1A, TMEM86B, IGLJ3, SDPR, NEURL1B, PLA2G2A, LOC100507311, EFEMP2, RNF135, ISG15, PIP5K1B, ZNF415, EMILIN1, PTPRB, GIMAP1, PPAP2C, AOC3, KCNJ10, NXPE3, ITGBL1, CKMT2, PPP2R5C, CD27, ENDOD1, PYROXD2, BCL11B, FHL2, NCAM1, PLA2G4A, IGK, ARHGAP25, ZEB2, RUNX3, SYT9, PNMAL1, PPP1R16B, FLJ32255, RBP1, SYTL2, TIMP3, LAMC3, PDGFD, DDX26B, SLC25A36, ITGB2-AS1, ETS1, EVA1C, ZNF160, CXCL16, CACNA2D1, NFATC1, DDR2, ADAMTS9-AS2, TMC8, GMFG, EIF5, FPR1, TMEM204, GALNT3, PARP8, GPR56, CCL4, PNOC, PIK3R5, ASGR1, WFDC1, RP11-38P22.2, NTN4, SGCD, TLR4, NEXN, PEG3-AS1, GSTM5, VSIG4, SLC1A2, MAP9, OAS1, XAF1, RAP1GAP2, TCEAL3, ITGA1, IFITM10, ZNF83, PRF1, AQP1, RBMS1, PBLD, CCR2, FAM46C, TGFBR3, FGB, SLC28A3, GIMAP6, CFH, KIAA1324L, BMS1P20, ZNF518B, TGM2, QSOX1, CLMP, PTGDR, C1QTNF7, SH3BP5, TRG-AS1, LEPR, DACT1, SLIT2, IL1RL1, IL10RA, NBEA, IFI27, FLI1, CD48, ANKRD36B, HLF, FAIM3, CXCR4, CECR1, KLF2, IL1RN, FOXQ1, ANKRD36, C16orf54, SELL, LAT, LAX1, CCR7, CDKN1A, SLC44A2, TIMP2, PDZRN4, RGS9, CCDC88C, VAV2, STK4, PEAR1, LBH, RAI2, SCML4, IDNK, LOC100505570, PVRIG, CSF2RB, LILRB2, STX11, IGHD, UBASH3A, PRKCQ-AS1, HCLS1, CMPK2, PRICKLE2, ZNF137P, FAM65B, TRBC1, EMR1, TGFB1I1, NSUN6, RNASE4, LGR6, AKNA, MAMDC2, C1orf162, PRKG1, TRAC, GNG2, WIPF1, TCF4, RCAN2, PIK3CD, PTN, RP11-642D21.1, C1QB, CFB, IRAK3, IP6K3, EMB, ACKR1, OLFML1, BACH2, AIM2, KRT19, SLC1A7, CSF1R, RUNX1T1, TLCD2, HSPA2, CD38, CD55, SLAMF7, C1QA, HOTS, DOCK2, CYSLTR1, SPON1, PIWIL4, EOMES, RASSF2, CYBRD1, CCND3, EML1, PTPRS, NLRC5, SLC16A14, SLC8A1, SERPINA1, TRAF3IP3, C8G, CLDN11, BLK, LOC100289058, SELPLG, IFI16, GPT2, PLEK2, PGR, IGFBP7, PTPLAD2, FRZB, TMEM173, SFMBT2, WFDC2, C4BPA, SLA, MCOLN2, C1QC, TNFSF11, ARGLU1, SPRY1, IFIT1, CCL21, AGPAT2, CELF2, PLAGL1, SSPN, FABP4, TPBG, TNFSF8, SLC31A2, NRXN3, NR2F1, PIK3CG, NLRC3, ZC3HAV1L, GBP5, CA5A, PRICKLE1, IGFBP6, RASGRP1, GLIPR1, FGD3, SRGN, CAMK4, RAC2, JAK3, THEMIS2, RORA, GLT8D2, ECM2, AEBP1, SMAD7, FGF1, CRIM1, CLEC7A, PBX4, CLEC11A, NCKAP1L, C1orf116, RORB, C2orf40, RAB31, ABCG5, TAL1, CSGALNACT1, ITIH3, DGAT2, P2RY14, ADRBK2, CDC42EP3, KLHL6, PRKCH, TNFSF13B, ADRA2A, ARHGDIB, ADCY7, KCNAB1, H1FX, HLA-DOA, GVINP1, CD53, P2RY8, CALD1, TLR8, ZNF468, GZMB, SH3RF3, RCSD1, GZMA, CHSY1, ANTXR1, LCK, INPP4B, LOXL4, TRAT1, P2RX5, FAM3B, ACSL5, SLC2A10, SELM, TCEA2, VIM, MYL9, MEG3, UBXN10, RP11-747H7.3, CLSTN1, SMIM3, ZNF677, GNB4, NR4A3, COL6A1, TAGLN, PTPRH, ZSCAN18, GPX3, EGR3, PSMB8-AS1, NTS, EFEMP1, TMEM71, CPA3, ARHGAP30, COL13A1, FMOD, MTHFD1, PAX5, LINC00342, LHFP, MX2, GPR65, MSR1, BANK1, SERINC2, PREX1, SH2D1A, MYOM1, KCTD12, S100A11, DPYSL3, LTB, ITGA4, LDHB, LINC00924, CD97, ALDH1A3, DOCK11, MAGEH1, CXCL5, COTL1, KAL1, CD52, SMOC2, MYH4, FMO3, CHST11, LXN, TDRP, AREG, ITGB2, VCAM1, AMPD1, TYROBP, GPR64, PNMA2, PPP1R14A, EVL, TMPRSS3, PMP22, CRTAM, DLGAP1-AS3, HCK, EVI2A, RASSF9, CTLA4, KLF7, CYBA, CD2, PDP1, ZNF559, SNX20, PROCR, THEMIS, ZKSCAN7, ZNF320, SLC22A17, MOXD1, RP11-305O6.3, CCND2, LY75, ARID5B, TSC22D3, CXCR6, MTHFD2, MMP2, DACT2, METRNL, LPAR1, ANXA13, DLK1, FAM43A, MFI2, HOXB3, TESC, RNASE6, MYRF, PLEK, BMPR1B, DNAJC12, PLAT, RGS18, ZNF331, AQP9, KIAA0226L, CORO1A, SERHL2, HLA-DPB1, OSBPL10, OGFRL1, IFI44, IFI44L, CXCL9, VLDLR, PLTP, TRPC1, MYOF, IQGAP1, SLC6A6, ALOX5, IGSF6, ARMCX1, WISP1, STON1, GABBR2, A2M-AS1, FPR3, PROM1, WT1, TMEM200A, HEPH, PMEPA1, RUNX2, DCDC2, UCP2, MSRB3, ACTA2, ABCB4, MGP, STMN2, SLC17A2, SPARC, ADSSL1, MYADM, COL6A3, DYNC2H1, LIMCH1, SNORD114-3, SYT13, ENPP5, OSMR, SERPINE2, LAPTM5, CLDN2, EPHA4 |
