# Supplementary material for: Microarray Data Mining and Preliminary Bioinformatics Analysis of Hepatitis D Virus-Associated Hepatocellular Carcinoma
Source: Biomed Res Int. 2021 Jan 30;2021:1093702. doi: 10.1155/2021/1093702 (PMC7867452; doi:10.1155/2021/1093702)
Supplement: Supplementary Materials — Table S1: DEGs from microarray datasets GSE55092 and GSE98383. Table S2: 948 DEGs related to HDV-associated HCC including 373 upregulated and 582 downregulated genes. Table S3: the five modules and contents obtained by WGCNA. [file 1093702.f1.zip › Table S3.docx]

| Table S3.The five modules and contents obtained by WGCNA | |
| --- | --- |
| Module Color | Gene name |
| blue | A2M-AS1, ABCB4, ABCG5, ACKR1, ACSL5, ACSM1, ADCY7, ADRA2A, ADSSL1, AFP, AKNA, ALOX5, AMPD1, ANKRD36BP2, ANTXR2, AQP9, ARHGAP25, ARHGAP30, ARHGDIB, ARID5B, ARMCX1, ATP8B4, BACH2, BANK1, BCL11B, BCL2L10, BLK, BST2, C16orf54, C1orf162, C1QA, C1QB, C1QC, CAMK4, CCDC88C, CCL21, CCL4, CCND3, CCR2, CCR7, CD163, CD2, CD27, CD38, CD48, CD52, CD53, CELF2, CHST11, CIART, CKMT2, CLDN2, CLEC7A, CMBL, CMPK2, COG3, COL4A3, COL4A4, COL5A3, CORO1A, COTL1, CPA3, CSF1R, CSF2RB, CST7, CTLA4, CXCL16, CXCL5, CXCL9, CXCR4, CXCR6, CYBA, CYP17A1, CYP7A1, CYSLTR1, DAB1, DGAT2, DOCK11, DOCK2, DYNC2H1, ECM2, EIF5, EMB, EOMES, EVI2A, EVL, FAM3B, FAM46C, FCRL5, FDPS, FGD3, FGF1, FLI1, FMO3, FOLR2, FPR1, FPR3, FTH1, GABBR2, GBP5, GIMAP1, GIMAP6, GMFG, GNG2, GPR65, GPT2, GREM1, GSN, GUCY2C, GVINP1, GZMA, GZMB, HCK, HCLS1, HIST1H2BC, HLA-DOA, HLA-DPB1, HLF, HMGCR, HMGCS1, HOMER1, HS3ST3B1, HSPA2, IDNK, IFI16, IFI27, IFI44, IFI44L, IFI6, IFIT1, IGLL5, IGSF6, IL10RA, IL1R2, IL2RB, IP6K3, IRF7, IRF9, ISG15, ITGA4, ITGB2, ITGB2-AS1, JAK3, KCNAB1, KCNJ10, KCTD12, KLB, KLHL6, KPNA3, LAPTM5, LAT, LAX1, LCK, LDHB, LEPR, LGALS3BP, LILRA2, LILRB2, LSS, LY75, LY9, MCOLN2, MCUR1, METRNL, MSR1, MTHFD1, MTHFD2, MX1, MYADM, MZB1, NCKAP1L, NLRC3, NLRC5, NR0B1, NRXN3, NSUN6, NTS, NXPE3, OAS1, OAS2, OLFML1, ONECUT2, OSBPL10, OSGIN1, P2RX5, P2RY14, P2RY8, PAX5, PBX4, PDP1, PDZRN4, PF4V1, PGR, PIK3CD, PIK3CG, PIK3R5, PLA1A, PLA2G2A, PLEK, PLEK2, PLTP, PNOC, PPP1R16B, PRDM1, PREX1, PRF1, PRKCB, PRKCH, PROCR, PRPSAP1, PSMB8-AS1, PTGDR, PTGR2, PTPRH, RAB38, RAC2, RASGRP1, RASSF2, RCSD1, RGS18, RHOH, RNASE6, RNF135, RNF213, RORA, RUNX3, SCLY, SCML4, SDPR, SELL, SELPLG, SERINC2, SFMBT2, SH2D1A, SIGLEC7, SIX1, SKIDA1, SLA, SLAMF7, SLC17A2, SLC1A2, SLC22A17, SLC30A10, SLC31A2, SLC8A1, SLC9A9, SNX20, SOAT2, SPATA41, SQLE, SRGN, STC2, STX11, SYT9, TAF4B, TCEA2, TCEAL3, TGFBR3, TGM2, THEMIS, THEMIS2, TLR4, TLR8, TMC8, TMEM100, TMEM144, TMEM173, TMEM71, TMEM86B, TMEM97, TMOD1, TNFRSF17, TNFSF11, TNFSF13B, TNFSF8, TRAF3IP3, TRAT1, TRG-AS1, TXNIP, TYROBP, UBASH3A, UBXN10, UCP2, VCAM1, VMO1, VSIG4, WFDC2, WIPF1, XAF1, XYLB, ZSCAN31 |
| brown | ADAMTS9-AS2, ANKRD18A, ANKRD36, ANKRD36B, ANXA13, AQP1, BACE2, BMPR1B, C14orf105, C1orf116, C2CD4A, CAV2, CD55, CERS6, COX7A1, CRIM1, CSGALNACT1, DCDC2, DDB2, DNAH14, DNER, DOCK5, ENDOD1, ENPP5, ERBB2, EVA1C, FAM83F, FAM84B, FMOD, GALNT3, GMNN, GPC4, GUSBP11, HKDC1, HOXB3, IL1RN, INPP4B, IQGAP1, KIFC2, KLF7, KRT19, LBH, LIMCH1, LOXL4, LTB, MAP2K6, MAP9, MATR3, MYOF, MYRF, NBEA, NFATC1, NME5, NOSTRIN, NTN4, OGFRL1, OSMR, P4HTM, PARP8, PDGFD, PDZK1IP1, PHTF1, PIWIL4, PMEPA1, PMFBP1, PNMA2, PNMAL1, PPP4R4, PRICKLE1, PROM1, PTGFR, PTPRS, PVRIG, QSOX1, RAP1GAP2, RASSF9, RNF150, S100A11, SCNN1A, SH3RF3, SIX4, SLC12A8, SLC25A36, SLC28A3, SLC44A2, SMAD6, SMAD7, SMIM24, SPOCK1, SPRR3, STON1, STX3, SULF1, SYT13, TDRP, TES, TESC, TFAP2A, TGM3, TGOLN2, TLCD2, TMED3, TNFAIP2, TVP23B, ZC3HAV1L, ZNF165, ZNF692 |
| green | AIM2, AREG, C1QTNF7, CCND2, CDC42EP3, CHSY1, CLEC11A, CLMP, CNRIP1, COL13A1, COL6A1, COL6A3, CRTAM, CYBRD1, DDR2, DNAJC12, DPYSL3, EMILIN1, EML1, EYA1, FHL2, FYN, GLIPR1, GLT8D2, GNB4, GP2, GRP, HEPH, IGFBP6, INSC, LPAR1, MAGEH1, MAMDC2, MFAP4, MGP, MMP2, MOXD1, NCAM1, P2RY12, PLA2G4A, PLA2R1, PLAGL1, PMP22, PPP2R5C, PRICKLE2, PTN, RAB31, RAI2, RBP1, RCAN2, RORB, RUNX2, SDC3, SERPINE2, SESN3, SFRP1, SH3BP5, SLC6A6, SLIT2, SMIM3, SSPN, STMN2, SYTL5, TIMP2, TMEM200A, TPBG, TSC22D3, VLDLR, WFDC1, WISP1 |
| grey | CCND1, KRT12, LOC100507477, SERHL2, UNC5D |
| turquoise | ABCF2, ACACA, AFAP1-AS1, AGFG1, AGPAT2, AIMP2, AKR1C1, ALPK3, ANKS1A, ANP32E, AP1M2, AP1S1, APTR, ARGLU1, ARHGEF37, ARPC1A, ASF1A, ASGR1, ASGR2, ASPH, ASRGL1, ATG10, ATP6V1C1, BAG2, BMS1P20, C4BPA, C4orf46, C7orf49, C8G, C8orf33, CA5A, CACNA1E, CACYBP, CBX3, CCDC150, CCDC93, CCNE1, CCT6A, CDC25B, CDC45, CDC6, CDCA5, CDCA7L, CDCA8, CDH12, CDKN1A, CENPE, CENPH, CENPI, CENPJ, CEP85, CFB, CFH, CHCHD3, CHEK2, CHML, CKS2, CLDN12, CLIP4, CLSTN1, CNIH4, CNPY2, COA1, COL28A1, COPS6, CSTA, CSTF3, DACH2, DANCR, DBF4, DCAF13, DDIAS, DDIT3, DENND2C, DFNA5, DIAPH3, DLAT, DLEU1, DLK1, DNAJB6, DNAJC3-AS1, DONSON, DPH3, DPH6, DPY19L1, DR1, DTYMK, EIF3B, EIF4EBP1, ENAH, ENPP6, ERC2, ESPL1, ETV1, EXO1, FABP4, FAM184A, FAM83H, FARP1, FBXL18, FGB, FHIT, FLAD1, FN1, FNBP1L, FOXQ1, FUCA2, GARS, GAS2L3, GEMIN6, GGCT, GLA, GNPDA1, GPX3, GPX7, H1FX, H2AFZ, HAGLR, HELLS, HENMT1, HILPDA, HINT3, HLTF, IFITM10, IL17D, IL17RB, IMMP1L, ITIH3, JAKMIP3, KCTD6, KHDRBS3, KIAA0895, KIAA1324L, KIAA1841, KIF18A, KIF23, KLHL13, KPNA2, LAMTOR4, LAPTM4B, LGR6, LIN28B, LINC00622, LOC101928820, LOC730101, LONRF1, LRIF1, LRP4, LSM4, MACROD2, MANEAL, MAP3K9, MBD1, MCM10, MCM4, MCM7, ME1, MEG3, METTL21A, MIS18A, MND1, MNS1, MPP6, MPP7, MRPS12, MS4A8, MSTO1, MTHFD2L, MX2, MYH4, MYOM1, MZT1, NAA20, NAA50, NCAPG2, NCAPH, NDC1, NDN, NDUFB9, NEIL3, NEU1, NEURL1B, NME1, NPM1, NR2F1, NRAS, NREP, NSMCE2, NUDT1, NUP155, NUP205, NUPR1, OGG1, OIP5, ORC6, OTUD6B, OTUD6B-AS1, PAFAH1B3, PARPBP, PBLD, PDCD2, PDSS1, PHEX, PIP5K1B, PKIB, PLK4, POLE2, POLQ, POLR3G, POPDC3, PPAT, PPM1E, PRMT6, PSMG3, PTDSS1, PTK2, RAB29, RAD51, RAD54B, RALA, RBM20, RECQL5, RFC3, RIT1, RNASE4, RNASEH2A, RNF187, ROBO2, RPL22L1, RRAGC, RRAS2, RYR3, SAP30, SARS, SASS6, SATB2, SCGN, SEC61A2, SERPINA1, SESTD1, SIPA1L2, SKP2, SLC16A10, SLC1A7, SLC28A2, SLC35G2, SLC40A1, SLC4A2, SMC4, SMKR1, SNORD114-3, SNX7, SPA17, SPAG5, SPHK1, SQSTM1, SRPK2, SSBP1, SSR1, SSX2IP, STK4, SV2B, SYT1, TACC3, TBC1D30, TET1, TEX11, TFRC, TIMELESS, TIPIN, TLCD1, TM4SF20, TMEM14A, TMPRSS3, TPD52, TRHDE, TRIB3, TRIM24, TRIM37, TRIM6, TRPC1, TSPAN7, TTC22, TTLL7, TYMS, TYW3, UBE2T, UGDH, UGT1A6, UNK, UPK3A, UTP15, VAV2, VSIG10L, WASF1, WDR12, WT1, XK, XPO4, XPOT, ZBED6CL, ZBTB41, ZFP82, ZKSCAN7, ZNF160, ZNF311, ZNF320, ZNF331, ZNF415, ZNF468, ZNF471, ZNF518B, ZNF559, ZNF57, ZNF677, ZNF697, ZNF83, ZSCAN18, ZSCAN9, ZWILCH |
| yellow | ACTA2, ALDH1A3, ANTXR1, AOC3, ARHGAP36, C2orf40, CACNA2D1, CALD1, CDC20B, COQ10A, CYGB, DACT1, DACT2, DDC, DLC1, DLGAP1-AS3, EFEMP1, EFEMP2, EGR3, ENG, EPHA4, ETS1, FAM124B, FAM19A5, FAM43A, FRY, FRZB, FXYD6, GALNT16, GFOD1, GGT5, GNA14, GRK5, GSTM5, IGFBP7, IL1RL1, ITGA1, ITGBL1, KLF2, LAMA2, LAMC3, LDB2, LHFP, LINC00924, LOC101927653, LTBP4, LXN, MSRB3, MYL9, NEXN, NFASC, NR4A3, NTF3, PCOLCE2, PDE2A, PEAR1, PLAT, PLCXD3, PPP1R14A, PRKG1, PTGDS, PTPRB, PTPRN2, RGS9, RPH3AL, RUNX1T1, SELP, SGCD, SLC16A14, SLC2A10, SMOC2, SPARC, SPON1, SPRY1, SULT1E1, SYTL2, TAGLN, TAL1, TBXA2R, TCF4, TGFB1I1, TIMP3, TMEM163, TMEM204, TMEM47, TMEM74, VAT1L, VIM, VSNL1, ZEB2 |

WGCNA, Weighted Gene Co-Expression Network Analysis.
